# EMT-activated secretory and endocytic vesicular trafficking programs underlie a vulnerability to PI4K2A antagonism in lung cancer

**DOI:** 10.1172/JCI165863

**Published:** 2023-04-03

**Authors:** Xiaochao Tan, Guan-Yu Xiao, Shike Wang, Lei Shi, Yanbin Zhao, Xin Liu, Jiang Yu, William K. Russell, Chad J. Creighton, Jonathan M. Kurie

**Affiliations:** 1 Department of Thoracic/Head and Neck Medical Oncology, The University of Texas–MD Anderson Cancer Center, Houston, Texas, USA.; 2 Department of Internal Medical Oncology, Harbin Medical University Cancer Hospital, Harbin, Heilongjiang Province, China.; 3 Department of Biochemistry and Molecular Biology, The University of Texas Medical Branch, Galveston, Texas, USA.; 4 Department of Medicine and Dan L Duncan Cancer Center, Baylor College of Medicine, Houston, Texas, USA.; 5 Department of Bioinformatics and Computational Biology, The University of Texas–MD Anderson Cancer Center, Houston, Texas, USA.

**Keywords:** Cell Biology, Oncology, Cancer gene therapy, Lung cancer, Oncogenes

## Abstract

Hypersecretory malignant cells underlie therapeutic resistance, metastasis, and poor clinical outcomes. However, the molecular basis for malignant hypersecretion remains obscure. Here, we showed that epithelial-mesenchymal transition (EMT) initiates exocytic and endocytic vesicular trafficking programs in lung cancer. The EMT-activating transcription factor zinc finger E-box–binding homeobox 1 (ZEB1) executed a PI4KIIIβ-to-PI4KIIα (PI4K2A) dependency switch that drove PI4P synthesis in the Golgi and endosomes. EMT enhanced the vulnerability of lung cancer cells to PI4K2A small-molecule antagonists. PI4K2A formed a MYOIIA-containing protein complex that facilitated secretory vesicle biogenesis in the Golgi, thereby establishing a hypersecretory state involving osteopontin (SPP1) and other prometastatic ligands. In the endosomal compartment, PI4K2A accelerated recycling of SPP1 receptors to complete an SPP1-dependent autocrine loop and interacted with HSP90 to prevent lysosomal degradation of AXL receptor tyrosine kinase, a driver of cell migration. These results show that EMT coordinates exocytic and endocytic vesicular trafficking to establish a therapeutically actionable hypersecretory state that drives lung cancer progression.

## Graphical abstract

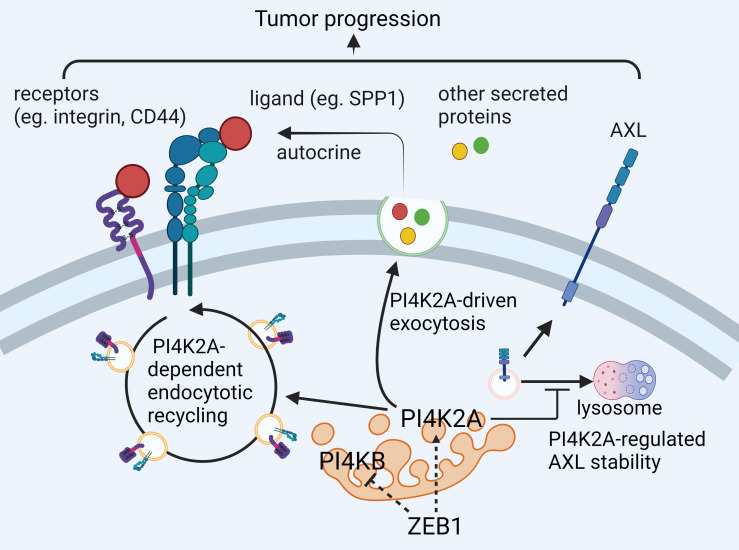

## Introduction

In a “tumor-as-organizer” hypothesis, metastatic propensity is determined by hypersecretory cancer cells that modify the extracellular matrix in ways that facilitate metastasis; for example, secreted extracellular matrix molecules and proteases enhance cancer cell invasion, cytokines negate antitumoral immunity, and growth factors sustain cancer cell viability (1). Hypersecretory states result in part from oncogenic mutations that activate secretory vesicle biogenesis in the Golgi, including p53 loss-of-function mutations and genetic amplification of Golgi-resident proteins involved in phosphatidylinositol (PI) metabolism (2–5). Conversely, extracellular cues emanating from the tumor microenvironment can activate prometastatic secretory programs in cancer cells (6), suggesting that malignant secretion can also be activated epigenetically. Therapeutic strategies designed to block malignant secretion based on a deep understanding of its molecular underpinnings warrant consideration.

In the conventional secretory pathway, proteins are transported as vesicular cargoes from the endoplasmic reticulum to the cell surface via the Golgi apparatus (7). In the Golgi, PI-4 phosphate (PI4P) is the membrane insertion site for Golgi phosphoprotein 3 (GOLPH3), which initiates secretory vesicle budding and release by creating F-actin bridges that exert tensile forces on Golgi membranes (8, 9). Golgi-resident PI4P is generated by the PI-4 kinases PI4KIIIβ (PI4KB) and PI4KIIα (PI4K2A) (10) and consumed by SAC1-like phosphatidylinositide phosphatase (SACM1L, also known as SAC1), a phosphoinositide phosphatase that resides in the Golgi and endoplasmic reticulum, creating a PI4P gradient across the Golgi stack (11). In contrast to PI4KB, which is primarily on the Golgi, PI4K2A localizes in both the trans-Golgi network and endosomal compartments and generates PI4P, which is essential for vesicle formation in the Golgi and receptor sorting on early and late endosomes (12, 13). Its dual role as a driver of secretory and endocytic vesicle trafficking is key in metastasis biology (3, 14).

Epithelial-mesenchymal transition (EMT) is an epigenetically regulated process by which cancer cells acquire metastatic activity and resistance to targeted therapeutic agents (15, 16). EMT-activating transcription factors (EMT-TFs) (e.g., ZEB and SNAIL families and basic helix-loop-helix transcription factors such as TWIST) govern large transcriptomes in part by silencing microRNAs (miRs) that target mRNAs encoding functionally diverse proteins, including EMT-activating transcription factors themselves, creating a feed-forward loop that drives EMT (17, 18). In lung cancer and other tumor types, the prometastatic activity of zinc finger E-box–binding homeobox 1 (ZEB1) resides largely in its capacity to silence miR-200, miR-182/-183, miR-34a, and miR-148a (19–23). Here, we postulated that EMT activates vesicular trafficking programs that drive malignant hypersecretion and metastasis in lung cancer.

## Results

### ZEB1 executes a PI4KB-to-PI4K2A dependency switch.

**Figure 1:**
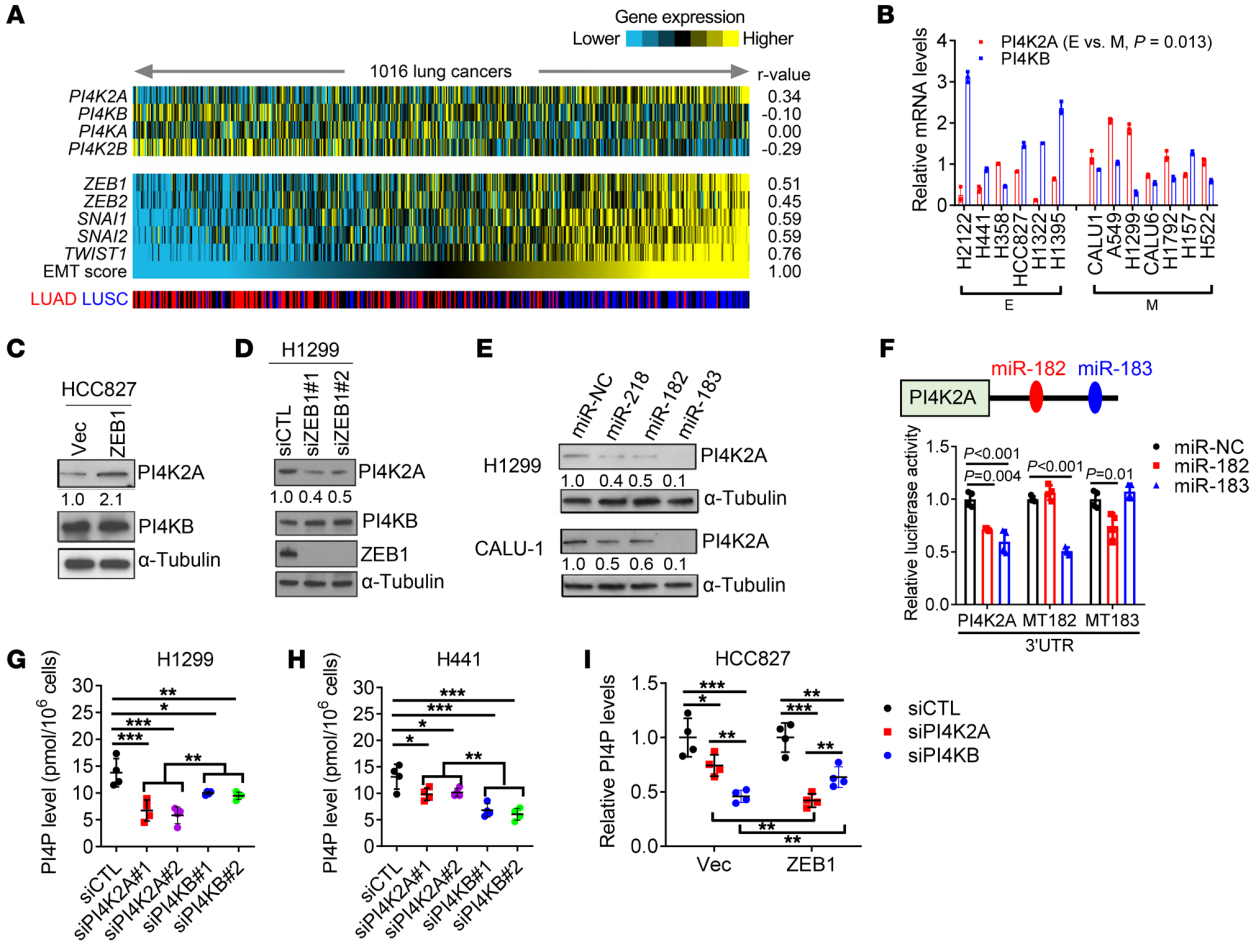
ZEB1 executes a PI4KB-to-PI4K2A enzymatic switch. (**A**) Heatmap illustration of mRNA expression levels in TCGA LUAD and LUSC cohorts (*n* = 1,016 tumors). An EMT score calculated for each tumor, as described previously (65), was correlated with each PI4K family member or, as a comparison, with the EMT-activating transcription factor using Pearson’s coefficient (*r* value). (**B**) qPCR analysis of *PI4K2A* and *PI4KB* mRNA levels in human lung cancer cell lines classified as epithelial (E) or mesenchymal (M). (**C** and **D**) WB analysis of PI4K2A, PI4KB, and ZEB1 levels in epithelial (**C**) or mesenchymal (**D**) cells subjected to ZEB1 gain or loss of function, respectively. Relative densitometric values are shown under the gel lanes. α-Tubulin was used as a loading control. Empty vector (Vec), scrambled control (siCTL), and ZEB1 (siZEB1) siRNAs were used. (**E**) WB analysis of PI4K2A in cells transfected with miR mimics. (**F**) PI4K2A 3′-UTR reporter assays. H1299 cells were cotransfected with miR mimics and reporters containing WT or miR-182/-183 binding site mutant 3′-UTRs (*n* = 4 replicates per condition). (**G**–**I**) PI4P ELISA in siRNA-transfected H1299 (**G**), H441 (**H**), and HCC827 (**I**) cells. Data indicate the mean ± SD from a single experiment incorporating biological replicate samples (*n* = 3, unless otherwise indicated) and are representative of at least 2 independent experiments. *P < 0.05, **P < 0.01, and ***P < 0.001, by 2-tailed Student’s *t* test for 2-group comparisons (**B**); 1-way ANOVA test for multiple comparisons (**F**–**I**). miR-NC, negative control mimic.

To test this hypothesis, we initially queried The Cancer Genome Atlas (TCGA) lung cancer cohort and found that an EMT-associated gene expression signature was positively correlated with PI4K2A levels, whereas other PI4K family members were either negatively correlated (PI4KB, PI4K2B) or not correlated (PI4KA) with EMT (Figure 1A). To investigate the mechanistic basis for PI4K2A’s positive correlation with EMT, we used a panel of human lung adenocarcinoma (LUAD) and lung squamous carcinoma (LUSC) cell lines that have been classified as epithelial or mesenchymal and in which high ZEB1 levels maintain a partial EMT (22, 24). In these cells, PI4K2A protein levels were higher in mesenchymal cells than in epithelial cells and were coordinately regulated in epithelial and mesenchymal cells subjected to ZEB1 gain or loss of function, respectively (Figure 1, B–D, and Supplemental Figure 1, A and B; supplemental material available online with this article; https://doi.org/10.1172/JCI165863DS1). Examination of the *PI4K2A* gene promoter region identified several E-boxes that could function as ZEB1-binding sites (Supplemental Figure 1C). To assess whether ZEB1 directly or indirectly increases *PI4K2A* gene transcription, we carried out RNA polymerase II (Pol II) ChIP assays on the *PI4K2A* gene promoter in ZEB1-deficient and -replete cells and found that ZEB1 depletion did not detectably alter RNA Pol II–binding activity (Supplemental Figure 1D), suggesting that ZEB1 increased PI4K2A levels through a posttranscriptional mechanism. The PI4K2A 3′-untranslated region (3′-UTR) contains a functional miR-218–binding site (25) and predicted binding sites for miR-182 and miR-183 (targetscan.org). Because miR-182 and miR-183 are known ZEB1 targets (16, 26) and correlate negatively with ZEB1 levels in TCGA LUAD cohort (Supplemental Figure 1E), we assessed whether PI4K2A is a target of ZEB1-silenced miRs. Indeed, miR-182 and miR-183 mimics decreased PI4K2A levels in H1299 and CALU-1 mesenchymal lung cancer cells and in HCC827_ZEB1, an epithelial LUAD cell line that has acquired mesenchymal properties owing to ectopic ZEB1 expression (Figure 1E and Supplemental Figure 1F) (19). Furthermore, miR-182 and miR-183 mimics suppressed the activity of reporters containing WT but not mutant PI4K2A 3′-UTRs lacking the predicted miR-182– or miR-183–binding sites (Figure 1F), suggesting that PI4K2A is a miR-182/-183 target and that ZEB1 relieves PI4K2A from miRNA silencing.

**Figure 2:**
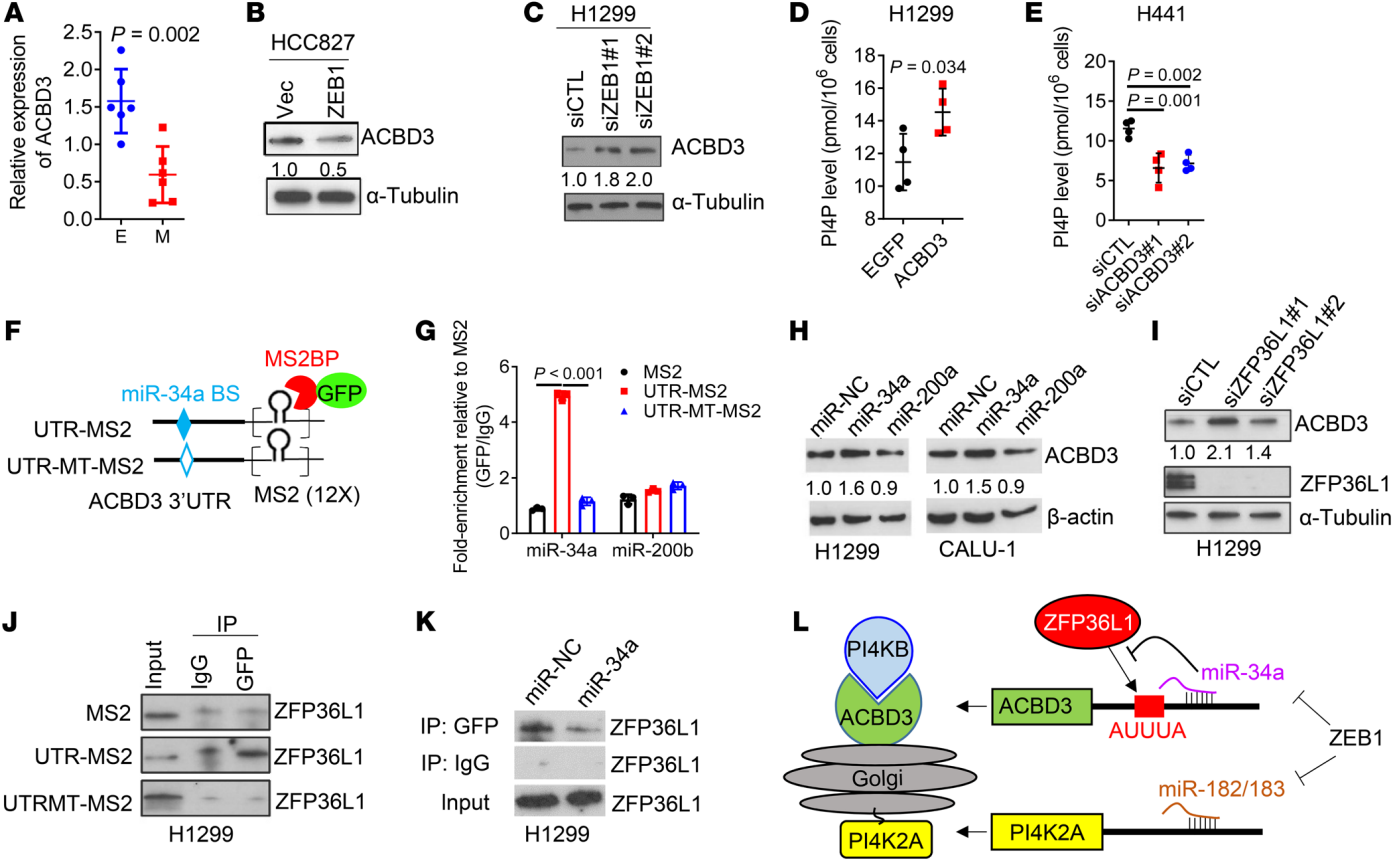
ZEB1 executes a PI4K2B-to-PI4K2A enzymatic switch. (**A**) qPCR analysis of *ACBD3* mRNA levels in the cell lines (dots) described in Figure 1B. (**B** and **C**) WB analysis of ACBD3 protein levels in epithelial (**B**) and mesenchymal (**C**) cells subjected to ZEB1 gain or loss of function, respectively. (**D** and **E**) PI4P ELISA in mesenchymal (**D**) and epithelial (**E**) cells subjected to ACBD3 gain or loss of function, respectively. (**F**) Schema showing constructs containing MS2 binding sites (12X) fused downstream of a WT or mutant (MT) ACBD3 3′-UTR lacking the miR-34a–binding site (BS). (**G**) MS2-based RIP. MS2-UTR–associated miR-34a was quantified as fold enrichment values relative to MS2. miR-200b was included as a negative control. (**H** and **I**) WB analysis of ACBD3 and ZFP36L1 levels in cells transfected with miR mimics (**H**) or siRNAs (**I**). (**J** and **K**) WB analysis of ZFP36L1 levels in cell lysates (input), an MS2-based RIP complex (GFP), or a negative control IP (IgG). (**L**) Schema of the working model. ZEB1 executes a PI4KB-to-PI4K2A dependency switch by silencing miR-34a and miR-182/-183. Data indicate the mean ± SD from a single experiment incorporating biological replicate samples (*n* = 3, unless otherwise indicated) and are representative of at least 2 independent experiments. *P* values were determined by 2-tailed Student’s *t* test for 2-group comparisons (**A**, **D**, and **E**); 1-way ANOVA test for multiple comparisons.

Because PI4K2A was unique among PI4K family members in its positive correlation with EMT (Figure 1A), we reasoned that the mesenchymal state may be dependent on PI4K2A for PI4P synthesis. To examine this possibility, we carried out siRNA-mediated depletion studies on PI4K2A in epithelial (H441, HCC827) and mesenchymal (H1299, HCC827_ZEB1) lung cancer cells. We compared the effects of depleting PI4K2A or PI4KB, which are both Golgi resident, but only PI4K2A is endosomal (10). Intracellular PI4P levels in mesenchymal cells decreased to a greater extent following depletion of PI4K2A compared with PI4KB depletion, whereas PI4P levels in epithelial cells decreased more sharply following depletion of PI4KB compared with depletion of PI4K2A (Figure 1, G–I, and Supplemental Figure 1, G–I). The reduced dependency of mesenchymal cells on PI4KB could not be explained by relative PI4KB levels (Figure 1, C and D), which led us to ask whether EMT alters the expression of PI4KB-associated proteins that are required for PI4KB enzymatic activity. The levels of the Golgi-resident scaffolding protein acyl coenzyme A binding domain–containing 3 (ACBD3), which tethers PI4KB to Golgi membranes (27), were higher in epithelial cells than in mesenchymal cells, were negatively correlated with ZEB1 levels in the Cancer Cell Line Encyclopedia (CCLE) (Figure 2A and Supplemental Figure 2, A and B), and were coordinately regulated in ZEB1 gain- and loss-of-function studies (Figure 2, B and C, and Supplemental Figure 1C). PI4P levels increased following ectopic ACBD3 expression in H1299 cells and decreased following siRNA-mediated ACBD3 depletion in H441 cells (Figure 2, D and E, and Supplemental Figure 2, D and E), supporting the conclusion that reduced ACBD3 levels underlay the switch to PI4K2A dependency in the mesenchymal state. To determine whether the PI4KB-to-PI4K2A dependency switch is regulated by other EMT-TFs, we carried out gain- and loss-of-function studies on SNAI1 and SNAI2 and found that they coordinately increased PI4K2A levels and decreased ACBD3 levels (Supplemental Figure 2, F–I), suggesting that the PI4KB-to-PI4K2A dependency switch was broadly linked to EMT.

To assess how ZEB1 silences ACBD3 expression, we initially carried out RNA Pol II ChIP assays on the *ACBD3* gene promoter in ZEB1-deficient and -replete lung cancer cells and found that, despite the presence of several E-boxes in the *ACBD3* gene promoter (Supplemental Figure 2J), ZEB1 depletion did not detectably alter RNA Pol II–binding activity (Supplemental Figure 2K), suggesting that ZEB1 silences ACBD3 through a posttranscriptional mechanism. Examination of the ACBD3 3′-UTR identified a predicted binding site for miR-34a, a ZEB1 transcriptional target (22). In MS2-based RNA immunoprecipitation (IP) assays, miR-34a mimics bound directly to WT but not mutant ACBD3 3′-UTRs lacking the miR-34a binding site (Figure 2, F and G), validating ACBD3 as a miR-34a target. ACBD3 mRNA and protein levels were increased by miR-34a mimics and decreased by miR-34a antagomirs (Figure 2H and Supplemental Figure 2, L–N). Although miRs typically silence target mRNAs, they can have the opposite effect if they block interactions with RNA-destabilizing proteins (28), a possibility supported by evidence that miR-34a mimics increased ACBD3 mRNA stability and the activity of ACBD3 3′-UTR reporters containing WT but not mutant miR-34a–binding sites (Supplemental Figure 2, O and P). A putative binding site for RNA-destabilizing proteins (AUUUA) (29) was identified next to the miR-34a–binding site and was found to be required for 3′-UTR reporter activation by miR-34a mimics (Supplemental Figure 2, Q and R). Among the RNA-destabilizing proteins identified in H1299 cells, ZFP36 ring finger protein–like 1 (ZFP36L1) played the most prominent role in ACBD3 destabilization (Figure 2I and Supplemental Figure 2, S–U). ZFP36L1 bound to the ACBD3 3′-UTR, and miR-34a mimics inhibited this binding (Figure 2, J and K). These findings support a model in which EMT-dependent microRNAs coordinate a PI4KB-to-PI4K2A dependency switch (Figure 2L).

### PI4K2A is a therapeutically actionable driver of lung cancer progression.

**Figure 3:**
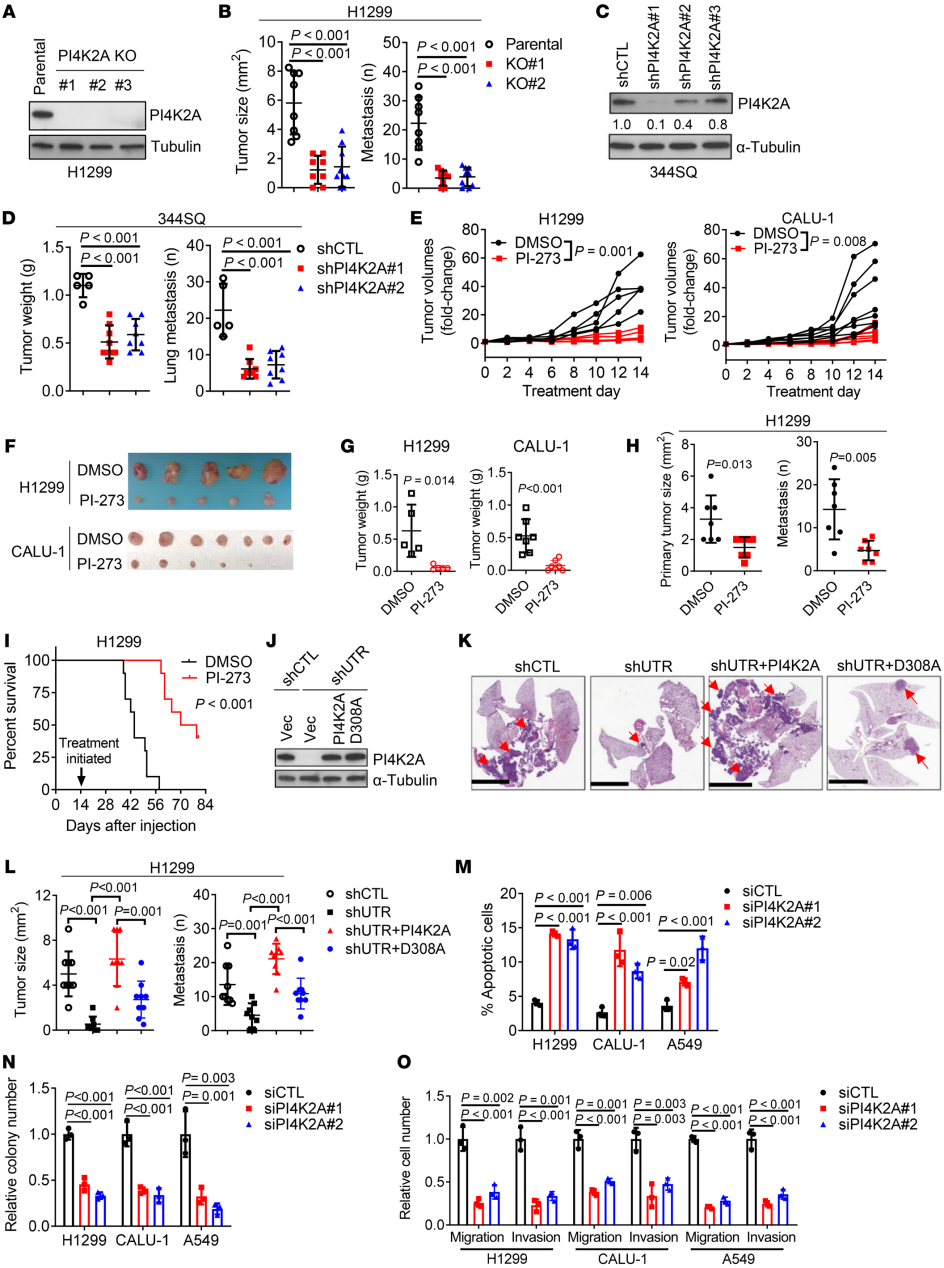
PI4K2A is a therapeutic target in mesenchymal lung cancer cells. (**A**) WB analysis confirming target gene deletion in PI4K2A-KO H1299 cells. (**B**) Orthotopic lung tumor size (left plot) and mediastinal and contralateral lung metastasis numbers (right plot) generated in nude mice (dots) by the intrathoracic injection of cells described in **A**. (**C**) WB analysis confirming target gene depletion in shRNA-transfected 344SQ cells. (**D**) Tumor weights (left plot) and lung metastasis numbers (right plot) generated in syngeneic, immunocompetent mice (dots) by subcutaneous injection of the cells described in **C**. (**E**) Daily subcutaneous tumor volume measurements (dots) in nude mice treated with PI-273 or vehicle (DMSO). (**F** and **G**) Tumor tissues removed at necropsy in **E** were imaged (**F**) and weighed (**G**). (**H**) Orthotopic lung tumor size (left plot) and metastasis numbers (right plot) in nude mice treated with PI-273 or vehicle. (**I**) Kaplan-Meier survival analysis of mice bearing orthotopic lung tumors treated with PI-273 or DMSO. (**J**) WB analysis demonstrating reconstitution of shPI4K2A-transfected H1299 cells (shUTR) with WT or enzyme-dead mutant (D308A) PI4K2A. Empty vector (Vec). (**K**) Orthotopic lung tumors (arrows) generated in nude mice by the cells in **J**. Scale bars: 5 mm. (**L**) Orthotopic lung tumor size (left plot) and metastasis numbers (right plot). (**M**) Annexin V/propidium iodide flow cytometric analysis of the apoptotic fraction in siRNA-transfected cells. (**N**) Colonies formed in soft agar by siRNA-transfected cells. Values are expressed relative to siCTL. (**O**) Boyden chamber migration and invasion assays on siRNA-transfected cells. Values are expressed relative to siCTL. Data indicate the mean ± SD from a single experiment incorporating biological replicate samples (*n* = 3, unless otherwise indicated) and are representative of at least 2 independent experiments. *P* values were determined by 2-tailed Student’s *t* test for 2-group comparisons (**E**–**H**); 1-way ANOVA test for multiple comparisons (**B**, **D**, and **L**–**O**; and log-rank test (**I**). shCTL, control shRNA.

In TCGA cohorts, PI4K2A mRNA levels are correlated with shorter survival durations in multiple tumor types, including LUSC, but not LUAD (Supplemental Figure 3A). Given that PI4K2A’s prognostic impact in non–small cell lung cancer (NSCLC) is histologic subtype specific, we assessed the biological role of PI4K2A in cell lines isolated from diverse NSCLC subtypes, including LUSC (CALU-1), large cell carcinoma (H1299), and LUAD (A549, H441, HCC827). In cell lines classified as mesenchymal (CALU-1, H1299, A549), PI4K2A inhibition by CRISPR/Cas9-mediated *PI4K2A* gene KO (Figure 3, A and B), shRNA-mediated PI4K2A depletion (Figure 3, C and D, and Supplemental Figure 3, B and C), or treatment with the PI4K2A small-molecule inhibitor PI-273 (30) (Figure 3, E–I, and Supplemental Figure 3D) reduced the growth and metastasis of tumors generated in mice. To investigate the mechanistic basis for these observations, we reconstituted PI4K2A-deficient H1299 cells with WT or kinase-dead mutant PI4K2A that localized in the Golgi similarly to endogenous PI4K2A (Supplemental Figure 3, E and F) and found that the antitumor effects of shRNA-mediated PI4K2A depletion were reversed only by reconstitution with WT PI4K2A (Figure 3, J–L), suggesting that the protumorigenic effects of PI4K2A were PI4P dependent. Furthermore, ectopic PI4K2A expression in epithelial H441 cells enhanced cell proliferation and tumor growth and metastasis in mice (Supplemental Figure 3, G–J), and PI4K2A depletion in mesenchymal lung cancer cells increased apoptosis in a monolayer culture, decreased colony formation in soft agar, and reduced migration and invasion in Boyden chambers (Figure 3, M–O, and Supplemental Figure 3, K–P). The effects of shRNA-mediated PI4K2A depletion were reversed by reconstitution with WT but not kinase-dead mutant PI4K2A (Supplemental Figure 3, Q and R) and were recapitulated by treatment with the PI4K2A small-molecule antagonists PI-273 or NC03 (30, 31), which exhibited greater activity in PI4K2A-replete than -deficient H1299 cells (Supplemental Figure 4) and in mesenchymal than epithelial lung cancer cells (Supplemental Figure 5, A–I). The sensitivity of epithelial HCC827 cells to PI4K2A antagonism was enhanced by ectopic ZEB1 expression (Supplemental Figure 5, J and K) or depletion of PI4KB or ACBD3 (Supplemental Figure 5, L–Q), supporting a connection between the PI4KB-to-PI4K2A dependency switch and vulnerability to PI4K2A antagonism.

### PI4K2A initiates a prometastatic secretory program in the Golgi.

**Figure 4:**
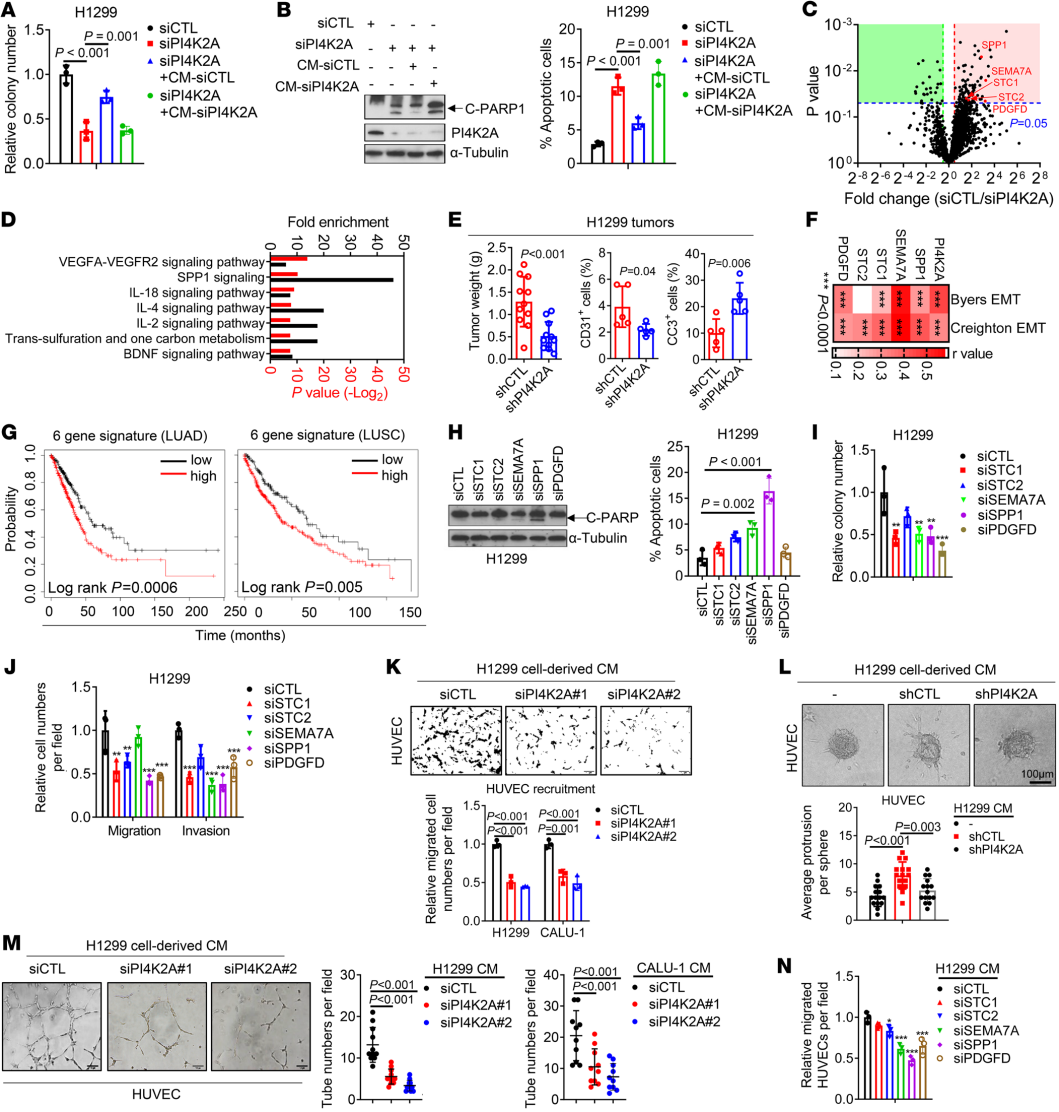
PI4K2A activates a prometastatic secretory program. (**A**) Relative soft agar colony numbers. siRNA-transfected H1299 cells were treated with CM samples from siRNA-transfected H1299 cells. (**B**) Apoptosis assays on siRNA-transfected H1299 cells treated with CM samples. WB analysis of cleaved PARP1 (C-PARP1) and PI4K2A (gel). (**C**) Volcano plot illustration of proteins (dots) identified by LC-MS analysis of CM samples. *P* value (*y* axis) and fold change (*x* axis) are shown. PI4K2A-upregulated secreted proteins (pink quadrant) of interest are labeled. (**D**) Gene Ontology analysis of the pink quadrant in **C**. (**E**) Subcutaneous tumors (dots) were weighed (left plot) and subjected to flow cytometry to quantify CD31^+^ cells (middle plot) and cleaved-caspase 3^+^ (CC3^+^) cells (right plot). (**F**) Heatmap illustration of the correlation between mRNAs and EMT scores (Byers or Creighton) in TCGA LUAD cohort. *r* values were determined by Pearson’s correlation. (**G**) Kaplan-Meier survival analysis of TCGA LUAD and LUSC cohorts based on 6-gene signatures of secreted proteins. Tumors were scored as being above (high) or below (low) each cohort’s median values. (**H**–**J**) Apoptosis assays and WB analysis of cleaved PARP1 (gel) (**H**), soft agar colony formation assays (**I**), and Boyden chamber migration and invasion assays (**J**) were carried out on siRNA-transfected H1299 cells. (**K**) HUVEC migration in Boyden chambers. CM samples from siRNA-transfected H1299 cells were loaded into the lower wells. Scale bars: 200 μm. (**L**) HUVEC spheroid invasion assay. HUVEC spheroids were seeded in 3D collagen and treated with CM samples. Scale bar: 100 μm. (**M**) HUVEC tube formation assay in 3D Matrigel following treatment with CM samples. Scale bars: 100 μm. (**N**) HUVEC migration in Boyden chambers. CM from siRNA-transfected H1299 cells was loaded into the lower chambers. Data indicate the mean ± SD from a single experiment incorporating biological replicate samples (*n* = 3, unless otherwise indicated) and are representative of at least 2 independent experiments. *P < 0.05, **P < 0.01, and ***P < 0.001, by 2-tailed Student’s *t* test for 2-group comparisons (**E**); 1-way ANOVA test for multiple comparisons (**A**, **B**, and **H**–**N**).

Based on our finding that PI4K2A depletion inhibited lung cancer progression, we speculated that PI4K2A activates a protumorigenic secretory process and initially addressed this possibility by performing conditioned medium (CM) transfer studies. Transfer of CM samples from PI4K2A-replete to PI4K2A-deficient H1299 cells partially rescued colony-forming activity in soft agar and reduced apoptosis in the monolayer culture, whereas CM samples from PI4K2A-deficient H1299 cells did not have this effect (Figure 4, A and B, and Supplemental Figure 6A). Proteomic analysis of CM samples from PI4K2A-deficient and -replete H1299 cells identified 92 proteins that were present at significantly higher concentrations in PI4K2A-replete cells (*P* < 0.05, fold change >1.5) (Figure 4C). By enrichment analysis, proteins downregulated in the CM from PI4K2A-deficient cells carried out proangiogenic, immunoregulatory, and prosurvival functions (Figure 4D). In line with these findings, PI4K2A-deficient tumors had reduced numbers of endothelial cells and increased numbers of apoptotic tumor cells (Figure 4E). The proteins identified included, among others, stanniocalcin 1 and 2 (STC-1/-2), semaphorin 7A (SEMA7A), osteopontin (SPP1), and platelet-derived growth factor D (PDGFD) (Figure 4C). Western blot (WB) analysis of CM samples confirmed that these proteins were reduced by siRNA-mediated PI4K2A depletion or PI-273 treatment (Supplemental Figure 6, B–D). Because high levels of several of the secreted proteins, singly and in combination, were found to correlate with EMT and shorter survival durations in TCGA lung cancer cohorts (Figure 4, F and G, and Supplemental Figure 6E), we performed in vitro assays on H1299 cells that were deficient or replete in the secreted proteins to assess their prometastatic roles. Depletion of several secreted factors recapitulated the effects of PI4K2A depletion on cell-intrinsic functions, such as apoptosis induction in monolayer culture, colony formation in soft agar, and migration and invasion in Boyden chambers (Figure 4, H–J, and Supplemental Figure 6, F–J). The abilities of human vascular endothelial cells (HUVECs) to migrate in Boyden chambers, invade in collagen gels, and generate tubes on Matrigel were enhanced by CM samples from PI4K2A-replete, but not -deficient, H1299 cells (Figure 3, K–M), and the chemotactic effects of H1299 cells on HUVECs were mediated in part by STC2, SEMA7A, SPP1, and PDGFD (Figure 4N and Supplemental Figure 6K).

**Figure 5:**
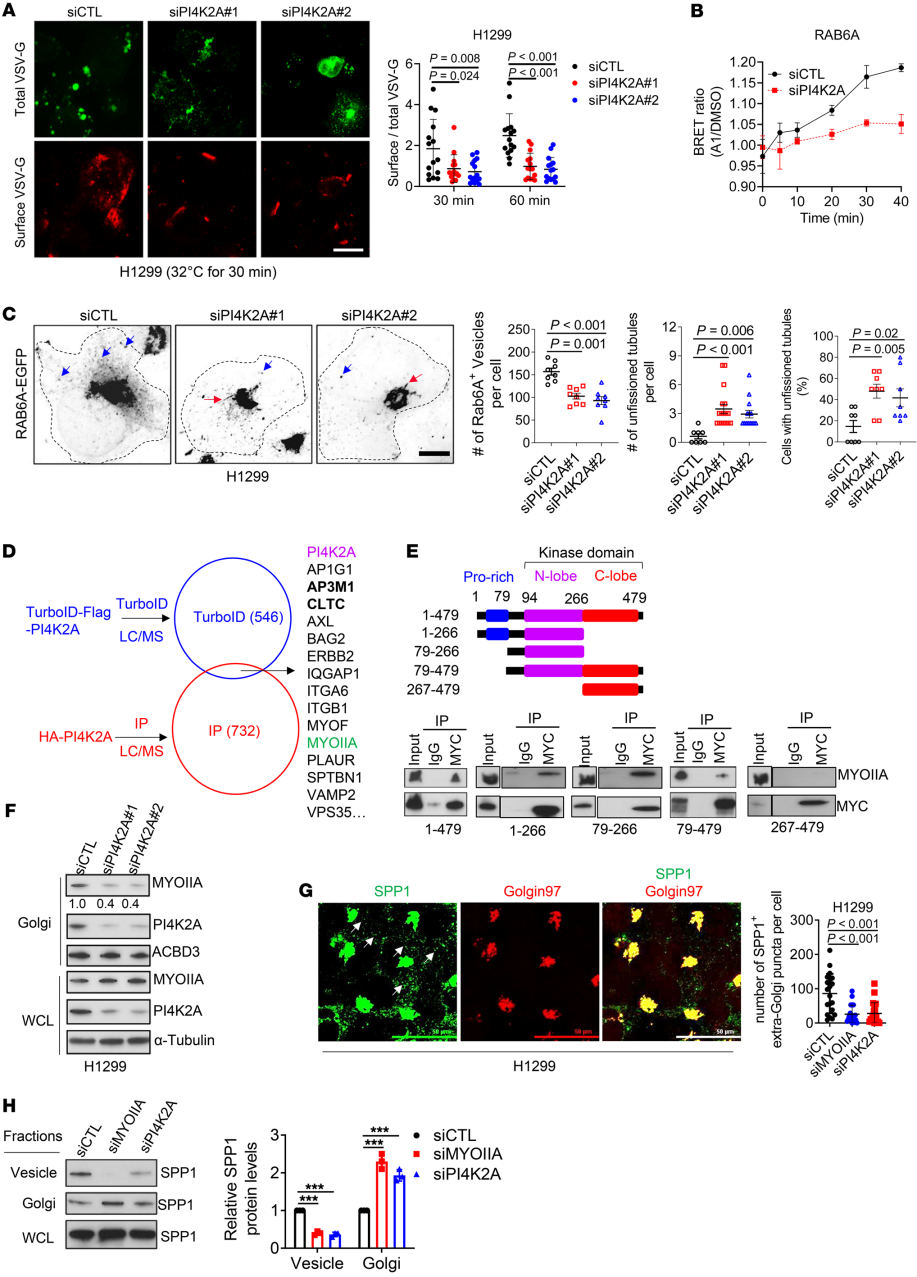
PI4K2A drives anterograde vesicular trafficking and promotes RAB6A^+^ vesicle fission. (**A**) Single-channel and merged confocal micrographs of total and surface VSV-G in H1299 cells cotransfected with siRNAs and EGFP–VSV-G and imaged 30 minutes after transfer to the permissive temperature. Plot shows the ratio of surface VSV-G to total VSV-G in each cell (dot) 30 or 60 minutes after transfer to 32°C. Scale bar: 20 μm. (**B**) BRET measurement of PI4P in RAB6A^+^ vesicles in siRNA-transfected H1299 cells. Results are expressed as a ratio of the values from GSK-A1–treated and vehicle-treated (DMSO) cells at each time point (*n* = 5 replicates per group). (**C**) Confocal micrographs of RAB6A^+^ vesicles (blue arrows) and unfissioned RAB6A^+^ tubules (red arrows) emerging from the Golgi. Scale bar: 20 μm. Dotted lines indicate the cell boundaries. Results were quantified per cell (dot plots). (**D**) Venn diagram of PI42KA-interacting proteins identified by TurboID and IP approaches. Overlapping proteins are listed on the right. Reported PI4K2A-interacting proteins (69, 70) are shown in bold. (**E**) Schematic illustration of full-length and truncated PI4K2A constructs. WB assays on whole-cell lysates (WCLs) (input) or IP proteins isolated from H1299 cells transfected with MYC-tagged PI4K2A constructs (gel). Full-length (1–479) and truncated constructs are indicated under the gels. IgG was used as the control IP. (**F**) WB analysis of WCLs (WCL) and Golgi-enriched fractions (Golgi) from siRNA-transfected H1299 cells. (**G**) Confocal micrographs of SPP1^+^ vesicles (arrows) in siRNA-transfected H1299 cells costained with anti-SPP1 and anti–Golgin 97 antibodies. Scale bars: 50 μm. Dot plot shows the vesicle numbers per cell. (**H**) WB analysis of WCLs or enriched subcellular fractions from siRNA-transfected H1299 cells. Densitometric values were normalized to siCTL (graph). Data indicate the mean ± SD from a single experiment incorporating biological replicate samples (*n* = 3, unless otherwise indicated) and are representative of at least 2 independent experiments. ***P < 0.001, by 2-tailed Student’s *t* test for 2-group comparisons (**B**); 1-way ANOVA test for multiple comparisons (**A**, **C**, **G**, and **H**).

Because PI4P serves as the Golgi membrane insertion site for GOLPH3 and other PI4P-binding proteins that initiate secretory vesicle biogenesis (4, 7, 9), we assessed the role of PI4K2A as a driver of anterograde vesicular trafficking. In temperature-sensitive mutant vesicular stomatitis virus G (VSV-G) assays performed on PI4K2A-deficient and -replete cells, PI4K2A deficiency retarded VSV-G transport to the plasma membrane (Figure 5A), an indication of impaired anterograde vesicular transport. In a bioluminescence resonance energy transfer (BRET) assay that quantifies PI4P levels in specific vesicle compartments (32), we found that siRNA-mediated PI4K2A depletion reduced PI4P levels in Ras-related protein RAB-6A–positive (RAB6A^+^) vesicles (Figure 5B), suggesting that PI4K2A was a source of PI4P in secretory vesicles. Furthermore, PI4K2A deficiency reduced RAB6A^+^ vesicle numbers and increased the numbers of unfissioned Golgi-associated RAB6A^+^ tubules (Figure 5C and Supplemental Figure 7A), an indication of impaired vesicle fission (33), suggesting that PI4K2A is essential for secretory vesicle biogenesis. Because PI4K2A lacks contractile and GTPase activities required for vesicle fission (9, 33), we reasoned that PI4K2A may form a complex with proteins that execute these functions. To address this possibility, we identified PI4K2A-associated proteins in pulldown assays on cells transfected with hemagglutinin-tagged (HA-tagged) PI4K2A and in proximity labeling assays on cells transfected with a PI4K2A/TurboID fusion construct (Figure 5D). From these 2 approaches, a total of 1,224 proteins were identified, 54 of which were identified by both approaches, including myosin heavy chain 9 (MYH9, also known as MYOIIA), a nonmuscle myosin that executes RAB6A vesicle fission in the Golgi (33). MYOIIA associated with the amino-terminal lobe of the PI4K2A kinase domain (Figure 5E), and Golgi localization of MYOIIA was reduced in PI4K2A-deficient cells (Figure 5F). As a readout for PI4K2A-dependent secretory vesicle biogenesis, we quantified SPP1-containing vesicle numbers in the cytoplasm and found that MYOIIA depletion reduced the numbers of SPP1-containing vesicles per cell similarly to PI4K2A depletion (Figure 5, G and H, and Supplemental Figure 7B), supporting a role for MYOIIA in PI4K2A-dependent secretory vesicle biogenesis.

### PI4K2A activates a prosurvival and promigratory vesicular trafficking program.

**Figure 6:**
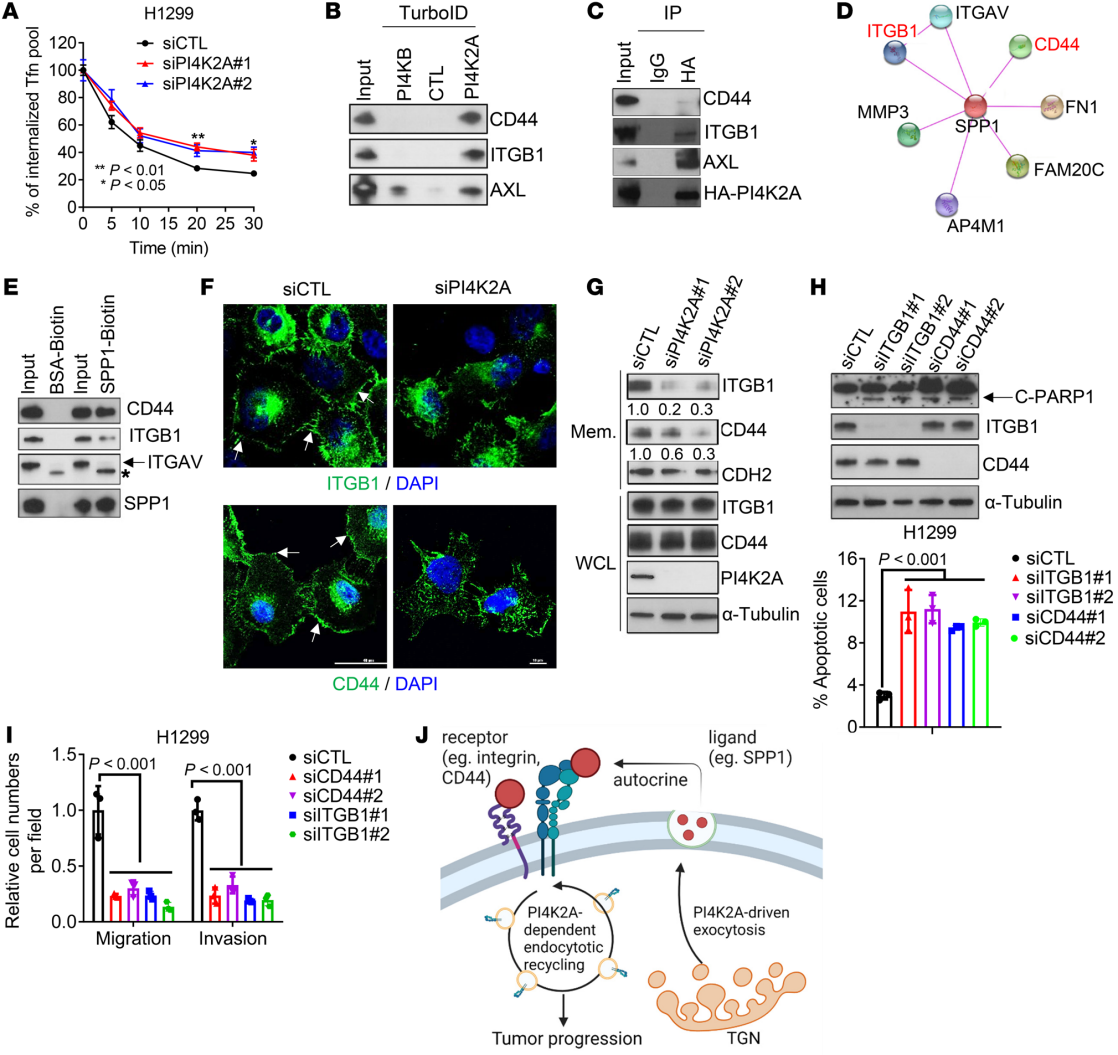
PI4K2A accelerates endocytic trafficking of SPP1 receptors. (**A**) Intracellular levels of biotinylated Tfn in siRNA-transfected H1299 cells were quantified at the indicated time points after a 30-minute pulse. The percentage of the internalized Tfn pool was calculated relative to the initial loading (*n* = 4 samples per condition). (**B**) WB analysis of CD44, ITGB1, and AXL protein levels in WCLs (input) or streptavidin bead–enriched protein samples from H1299 cells that were transfected with PI4K2A/TurboID construct and treated with biotin. Controls included the PI4KB/TurboID construct (PI4KB) and Turbo alone (CTL). (**C**) WB analysis of WCLs (input) or anti-HA immunoprecipitates from H1299 cells transfected with HA-tagged PI4K2A. IgG was used as the control IP. (**D**) SPP1 protein-protein interaction network (STRING-db.org). (**E**) WB analysis of proteins isolated by streptavidin bead–based pulldowns carried out on H1299 cells treated with biotin-labeled recombinant SPP1. (**F**) Confocal micrographs of plasma membrane–bound ITGB1 (arrows, upper panels) and CD44 (arrows, lower panels) in nonpermeabilized siRNA-transfected H1299 cells stained with antibodies against endogenous ITGB1 or CD44. Scale bars: 10 μm. (**G**) WB analysis of cell membrane–enriched fractions (Mem.) and WCL. Densitometric values are shown under the gels. (**H**) WB analysis of cleaved PARP1 (gel) and flow cytometric analysis of annexin V/PI–stained cells (graph) to quantify apoptosis in siRNA-transfected H1299 cells. (**I**) Boyden chamber migration and invasion assays on siRNA-transfected cells. (**J**) Schematic illustration of the working model. PI4K2A coordinates exocytic and endocytic vesicular trafficking to activate an SPP1-dependent autocrine loop. Data indicate the mean ± SD from a single experiment incorporating biological replicate samples (*n* = 3, unless otherwise indicated) and are representative of at least 2 independent experiments. *P* values were determined by 1-way ANOVA test for multiple comparisons (**A**, **H**, and **I**).

Given that PI4K2A localizes in endosomes (Supplemental Figure 8A) and facilitates endosomal trafficking (13, 34), we assessed PI4K2A as a mediator of ZEB1-dependent endosomal recycling, which has been shown to establish a polarity axis in lung cancer cells (14). We treated PI4K2A-deficient and -replete cells with biotin-labeled transferrin (Tfn), which binds to the Tfn receptor and is internalized and transported back to the plasma membrane via the endocytic recycling pathway (35). Although intracellular Tfn measurements at 5-minute intervals after initiating endocytosis demonstrated no evidence that PI4K2A regulates Tfn internalization (Supplemental Figure 8B), measurements begun 30 minutes after endocytosis initiation demonstrated higher Tfn levels in PI4K2A-deficient cells (Figure 6A), an indication of delayed endocytic trafficking.

To confirm that PI4K2A drives endocytic trafficking and to identify a biological role for PI4K2A-dependent endocytic trafficking in lung cancer, we identified PI4K2A-associated cell-surface receptors in our TurboID proximity ligation assays (Supplemental Table 1) and then assessed how PI4K2A influences the intracellular fates of those receptors. Among the 15 cell-surface receptors identified by TurboID (Supplemental Table 1), CD44 molecule and integrin-β1 (ITGB1) were of interest because they are receptors for SPP1 (36, 37) and undergo endocytosis and are recycled back to the plasma membrane with defined kinetics (38, 39). We confirmed that CD44 and ITGB1 are PI4K2A-associated proteins (Figure 6, B and C, and Supplemental Figure 8C) and that SPP1 binds to CD44 and ITGB1 in mesenchymal lung cancer cells (Figure 6, D and E). To assess whether PI4K2A influences recycling of CD44 and ITGB1, we quantified plasma membrane–bound CD44 and ITGB1 levels in PI4K2A-deficient and -replete cells and found reduced levels in PI4K2A-deficient H1299 cells, whereas total intracellular levels were not affected (Figure 6, F and G, and Supplemental Figure 8D), suggesting that PI4K2A deficiency delayed endosomal recycling of ITGB1 and CD44. Furthermore, siRNA-mediated depletion of ITGB1 or CD44 recapitulated the effect of SPP1 depletion in apoptosis and cell migration assays (Figure 6, H and I, and Supplemental Figure 8, E and F), and recombinant SPP1 protein treatment enhanced the migratory and colony-forming activities of SPP1-deficient, but not ITGB1- or CD44-deficient, cells (Supplemental Figure 8, G–K). Thus, PI4K2A activated an SPP1-dependent autocrine loop by coordinately enhancing ligand secretion and receptor recycling (Figure 6J).

**Figure 7:**
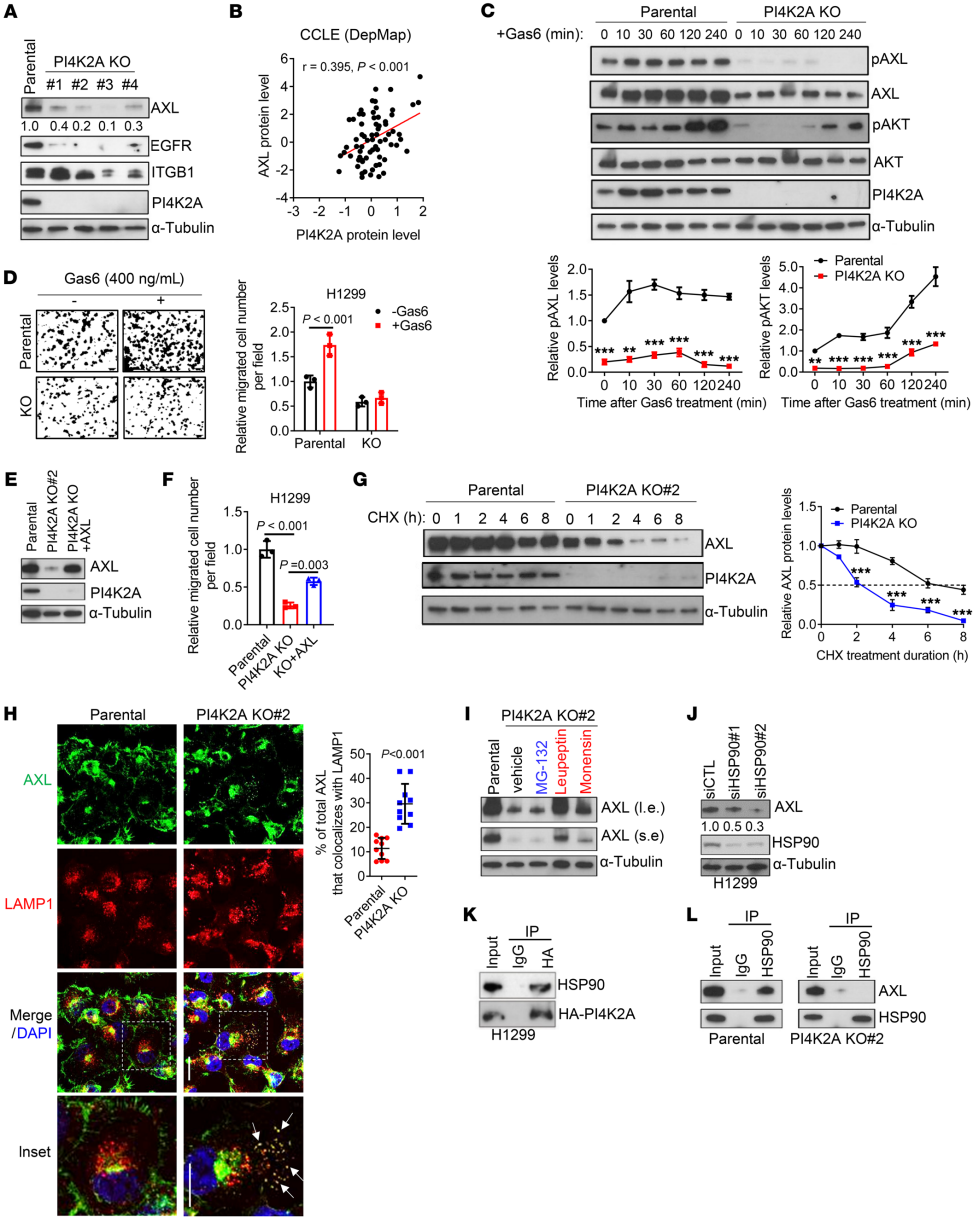
PI4K2A stabilizes AXL. (**A**) WB analysis of parental and PI4K2A-KO H1299 cells. Densitometric values are shown under the gels. Positive (EGFR) and negative (ITGB1) controls. (**B**) Correlation between AXL and PI4K2A protein levels in cell lines (dots). (**C**) WB analysis of parental and PI4K2A-KO H1299 cells treated with the AXL ligand Gas6. pAKT, phosphorylated AKT. Graphs show densitometric analysis of pAXL and pAKT levels normalized to *t* = 0 minutes. (**D**) Boyden chamber migration assays on parental and PI4K2A-KO H1299 cells treated with (+) or without (–) Gas6. (**E**) WB analysis of parental and PI4K2A-KO H1299 cells stably transfected with empty vector or AXL. (**F**) Boyden chamber migration assays on cells in **E**. (**G**) WB analysis of parental and PI4K2A-KO H1299 cells treated with cycloheximide (CHX). Graphs show the densitometric values. (**H**) Single-channel and merged confocal micrographs of parental and PI4K2A-KO cells costained with anti-AXL and anti-LAMP1 antibodies. Lysosomal AXL (inset, arrows) was quantified as the percentage of total AXL that colocalized with LAMP1 per field (*n* = 10 fields per condition). Scale bar: 10 μm. Original magnification, ×2.5 (enlarged insets). (**I**) WB analysis of PI4K2A-KO cells treated with proteasomal (MG132) or lysosomal (leupeptin or monensin) inhibitors. DMSO was used as the vehicle. l.e., long exposure duration; s.e., short exposure duration. (**J**) WB analysis of siRNA-transfected H1299 cells. Densitometric values are shown under the gel. (**K**) WB analysis of WCLs (input) and anti-HA immunoprecipitates from H1299 cells transfected with HA-tagged PI4K2A. IgG was used as the negative control IP. (**L**) WB analysis of WCLs (input) and anti-HSP90 immunoprecipitates from parental and PI4K2A-KO H1299 cells. IgG was used as the negative control IP. Data indicate the mean ± SD from a single experiment incorporating biological replicate samples (*n* = 3, unless otherwise indicated) and are representative of at least 2 independent experiments. **P < 0.01 and ***P < 0.001, by 2-tailed Student’s *t* test for 2-group comparisons (**C**, **D**, **G**, and **H**); 1-way ANOVA test for multiple comparisons (**F**).

A third receptor of interest in our TurboID database is AXL receptor tyrosine kinase (AXL), which plays a key role in lung cancer progression and is transported through endosomal recycling and lysosomal transport pathways with defined kinetics (40). To assess how PI4K2A influences the intracellular fate of AXL, we initially quantified AXL protein levels in parental and PI4K2A-KO cells and found that AXL protein levels were sharply reduced in PI4K2A-KO cells (Figure 7A). In line with this finding, AXL protein levels are positively correlated with PI4K2A levels in the CCLE lung cancer cell line cohort (Figure 7B). Supporting a functional linkage between PI4K2A and AXL, PI4K2A deficiency impaired ligand-induced, AXL-dependent pathway activation and cell migration (Figure 7, C and D, and Supplemental Figure 9, A and B), and AXL gain- and loss-of-function studies showed that AXL was a key effector of PI4K2A in cell migration assays (Figure 7, E and F, and Supplemental Figure 9, C–E). PI4K2A deficiency did not detectably alter *AXL* mRNA levels (Supplemental Figure 9F) but accelerated AXL protein degradation (Figure 7G) and increased lysosomal localization of AXL protein (Figure 7H). Lysosome inhibitor treatment increased AXL protein levels in PI4K2A-deficient cells (Figure 7I). Thus, PI4K2A enhanced AXL protein stability.

Because PI4K2A localizes to Ras-related protein RAB-7A–positive (RAB7A^+^) vesicles (32), which can rescue cargoes from lysosomal degradation by redirecting them from late endosomes to other compartments (41), we reasoned that PI4K2A may enhance AXL protein stability by generating PI4P in the RAB7A^+^ vesicle compartment. In support of this possibility, AXL colocalized with RAB7A (Supplemental Figure 9G), and siRNA-mediated PI4K2A depletion reduced PI4P levels in RAB7A^+^ vesicles (Supplemental Figure 9H). However, AXL protein levels did not change following PI-273 treatment (Supplemental Figure 9I), and AXL protein levels in PI4K2A-KO cells were rescued by reconstitution with WT or enzyme-dead mutant PI4K2A (Supplemental Figure 9J), suggesting that PI4K2A enhances AXL protein stability through a kinase-independent mechanism. Heat shock protein 90 (HSP90), which enhances AXL stability (42), was identified as a PI4K2A-interacting protein (Figure 7, J and K, and Supplemental Table 1), and HSP90 interacted with AXL in parental but not PI4K2A-KO H1299 cells (Figure 7L), suggesting that PI4K2A may stabilize AXL by recruiting HSP90.

## Discussion

Metastatic cancer cells are resistant to currently available therapeutic approaches and are the foremost cause of cancer-related death (43). Because cancer cells acquire metastatic properties and therapeutic resistance through transcriptional programs activated by EMT (44), strategies to selectively target mesenchymal cancer cells warrant consideration. The findings presented here identify PI4K2A as an EMT-dependent therapeutic vulnerability.

In a “tumor-as-organizer” hypothesis (1), the principal orchestrators of a metastasis-permissive tumor microenvironment (TME) are cancer cells themselves. Factors emanating from hypersecretory cancer cells activate autocrine loops that maintain cancer cell survival and chemoattract a variety of stromal cell types that create an immunosuppressed, fibrotic, and vascularized TME (45). Hypersecretory states in cancer cells are initiated by commonly occurring oncogenic mutations and chromosomal amplifications that drive neoplastic transformation (2–4, 9, 46, 47), which is the basis for the current belief that hypersecretion is a cell-intrinsic process (48). However, evidence presented here suggests that cancer cells gain hypersecretory features by undergoing EMT, a process initiated by extracellular cues emanating from the TME (49), which raises the possibility that, once activated by oncogenic mutations, the TME-organizing capacity is reinforced by extracellular cues, completing a feed-forward loop involving bidirectional crosstalk between cancer cells and the TME in which they reside.

miRs are a class of noncoding RNAs that fine-tune the expression of target genes involved in multiple prometastatic biological processes, including EMT, invasion, and immunosuppression (50). Although miRs typically silence target genes through recruitment of the miR-induced silencing complex (51), which induces translation inhibition or mRNA decay, miRs can also increase target gene mRNA levels by binding to regulatory elements in 5′-UTR promoter sequences or by interfering with RNA-destabilizing proteins that bind to 3′-UTR elements (28, 52, 53). Here, we show that miR-34a regulates PI4KB activity through this mechanism and that ZEB1 silences miR-34a and miR-182/-183 to coordinately inactivate PI4KB and increase PI4K2A levels, thereby executing a PI4KB-to-PI4K2A dependency switch that drives lung cancer progression.

PI4P is a major regulator of vesicular trafficking and modulates intracellular signaling and metabolic pathways (54). PI4P is generated in part by 4 mammalian PI4K enzymes, including 2 type II kinases (PI4K2A and PI4K2B) and 2 type III kinases (PI4KA and PI4KB) that reside in distinct subcellular compartments (12). High levels of PI4KB, PI4KA, and PI4K2A are associated with a poor prognosis in multiple tumor types and drive malignant progression through distinct mechanisms (3, 55–57). A chromosome 1q21.3 amplicon harbors PI4KB and maintains cancer cell survival by activating a PI4KB-dependent secretome (3). PI4K2B, however, functions as a negative regulator of cancer cell invasion and is frequently deleted or downregulated in human cancers (58). PI4K2A maintains cancer cell survival and promotes tumorigenesis by enhancing EGFR protein stability (59, 60) and by facilitating trafficking of misfolded proteins to the lysosome (61). Here, we show that PI4K2A stabilizes AXL and coordinates exocytic and endocytic trafficking of SPP1 and its receptors, respectively, to activate a protumorigenic autocrine loop in mesenchymal lung cancer cells.

Given the findings reported here and elsewhere (3, 5), secretory blockade warrants consideration as a targeted therapeutic strategy in cancer. PI4KB small-molecule inhibitors induce apoptosis in 1q21.3-amplified, but not diploid, cancer cells (3, 25). Similarly, p53 loss drives a secretory process that maintains the survival of p53-deficient cancer cells and can be targeted using existing small-molecule antagonists (2, 5). Here, we show that an EMT-induced enzymatic switch creates a vulnerability to PI4K2A antagonists. Thus, commonly occurring oncogenic mutations and epigenetic processes drive addictive secretory processes that are therapeutically actionable.

## Methods

### Mice.

129/sv mice were bred and maintained in the Research Animal Support Facility at The University of Texas MD Anderson Cancer Center; nude mice were purchased from The Jackson Laboratory. Mice underwent standard care and were euthanized at predetermined time points or at the first signs of morbidity according to the standards set forth by the IACUC. To generate subcutaneous tumors, syngeneic, immunocompetent mice (*n* = 7–10 mice per group) or nu/nu mice (*n* = 5–10 mice per group) were subcutaneously injected with 1 × 10^6^ murine or human lung cancer cells, respectively. To generate orthotopic lung tumors, nu/nu mice were intrathoracically injected with 1 × 10^6^ human lung cancer cells. When indicated, mice were treated for 2–4 weeks with PI4K2A inhibitor PI-273 (25 mg/kg/day) or vehicle intraperitoneally beginning 2 weeks after tumor cell injection. The mice were monitored daily for signs of morbidity and necropsied at week 5 (for murine cells) or week 6 (for human cells). Subcutaneous tumors were measured every 2 days and weighed at necropsy. Primary lung tumors and lung metastases were measured and counted and confirmed by H&E staining.

### Cell lines.

Human lung cancer cell lines (A549, H1299, H157, CALU-1, CALU-6, H322, H441, H358, HCC827, H522, H2122, H23, H1792, H441, H358, H226, and H1395) and HUVECs were purchased from the American Type Culture Collection (ATCC). HCC827-Vec and HCC827-ZEB1 cells and the murine LUAD cell line 344SQ were previously described (19). Lung cancer cells were cultured in RPMI 1640 containing 10% FBS. HUVECs were cultured in EGM-2 Endothelial Cell Growth Medium (Lonza). Cells were maintained at 37°C in an incubator with a humidified atmosphere containing 5% CO_2_. Cells were transfected with jetPRIME Versatile DNA/siRNA transfection reagent (Polyplus). Stable cell transfectants were selected using puromycin (for pLVX and pLKO.1 vectors) or G418 (for pcDNA3.1 and pEGFP-C3 vectors). PI4K2A-KO H1299 cells were generated using the CRISPR/Cas9 system in the Cell-Based Assay Screening Service Core Facility at Baylor College of Medicine (guide RNA sequences: 5′-CCCACTAGTGTCCCCCGAGC-3′ and 5′-TTTCCCGAGCGCATCTACCA-3′).

### Reagents.

We purchased SYBR Green, FBS, RPMI 1640, Alexa Fluor–tagged secondary antibodies, and DAPI from Life Technologies (Thermo Fisher Scientific); paraformaldehyde from Electron Microscopy Sciences; puromycin from InvivoGen; G418 from Corning; brefeldin A (B7651) from MilliporeSigma; murine (TRCN0000025579, TRCN0000025580, and TRCN0000025582) and human (TRCN0000196550) PI4K2A shRNAs from MilliporeSigma; PI4KB (SASI_Hs01_00149544),PI4K2A (SASI_Hs01_00190417 and SASI_Hs01_00190418), ZEB1 (SASI_Hs02_00330526 and SASI_Hs02_00330527), SNAI1 (SASI_Hs01_00039785 and SASI_Hs01_00039786), SNAI2 (SASI_Hs01_00159363 and SASI_Hs01_00159364) siRNAs from MilliporeSigma; antibodies against α-tubulin (no. T9026), Flag tag (no. F3165), MYC tag (no. 05-724), or EGFP (no. G6539) from MilliporeSigma; antibodies against PARP-1 (no. 9542), HA-tag (nos. 3724 and 2367), β-actin (no. 4970), GM130 (no. 12480), Golgin-97 (no. 13192), AXL (no. 8661), and EGFR (no. 2256) from Cell Signaling Technology; antibodies against ACBD3 (sc-101277), ZEB1 (sc-25388), and PI4K2A (sc-390026) from Santa Cruz Biotechnology; antibodies against SPP1 (22952-1-AP), SEMA7A (18070-1-AP), HSP90 (11405-1-AP), ZFP36L1 (12306-1-AP), and CD44 (15675-1-AP) from Proteintech; antibodies against ITGB1 (GTX636657) from GeneTex; antibodies against RNA polymerase II (AB_2732926) from Active Motif; recombinant SPP1 proteins (ab92964) from Abcam; recombinant Gas6 (885-GSB) and SPP1 (1433-OP-050) proteins from R&D Systems; PI-273 (HY-103489) from MedChemExpress; NC03 (AOB17420) from Aobious; and miRNA mimics (HMI0508, HMI0275, and HMI0280) and control mimics (HMC0002) from MilliporeSigma. PI4K2A antibody for immunofluorescence analysis was a gift from Pietro De Camilli (Yale School of Medicine, New Haven, Connecticut, USA) (62).

### Vector construction.

The human PI4K2A and SNAI2 coding sequences were isolated by performing PCR on cDNA prepared from human H1299 cells and cloned into pcDNA3.1 or pEGFP-C3. To generate 3′-UTR reporters, the PI4K2A and ACBD3 3′-UTR sequences were amplified by PCR and inserted downstream of *Renilla* luciferase or the EGFP cassette. To generate the TurboID-PI4K2A construct, PI4K2A coding sequences (a gift from Feng-Qian Li and Ken-Ichi Takemaru, Addgene plasmid 74223) were inserted into the Flag-TurboID-pcDNA3 vector using XhoI and XbaI. pSL-MS2-12X was double-digested with EcoRI and XhoI, and the MS2-12X fragment was subcloned into the pcDNA3.1 to make the pcDNA-MS2 (12X) vector. ACBD3 3′-UTR was inserted upstream of the MS2 sequence in the pcDNA-MS2 (12X) vector. pMS2-GFP (Addgene plasmid 27121) and pSL-MS2-12X (Addgene plasmid 27119) were gifts from Robert Singer (Albert Einstein College of Medicine, Bronx, New York, USA) (63). AXL-MYC-pcDNA3.1 was a gift from Rosa Marina Melillo (University of Naples Federico II, Naples, Italy) (Addgene plasmid 105932). GFP-PH-FAPP1 was a gift from Tamas Balla and David Bryant (NIH, Bethesda, Maryland, USA; Addgene plasmid 161986) (64). SNAI1-HA-pcDNA3.1 was a gift from Paul Wade (National Institute of Environmental Health Sciences, Durham, North Carolina, USA) (Addgene plasmid 31697). Mutations were introduced by PCR. The PCR primers utilized are listed in Supplemental Table 2.

### PI4P ELISA.

Intracellular PI4P levels were determined using the PI4P Mass ELISA Kit (K-4000E, Echelon Biosciences) as previously described (3).

### Cell proliferation, colony formation, and apoptosis assay.

Cell density assays on plastic were performed using Cell Proliferation Reagent WST-1 (Roche). For colony formation assays, cells were plated on plastic (800 cells per well) for 10 days or in soft agar (5 × 10^4^ cells per well) for 10–14 days, and colonies were stained with crystal violet. Apoptosis was determined by WB analysis of cleaved PARP1 or by flow cytometry using the Dead Cell Apoptosis Kit (V13242, Thermo Fisher Scientific) according to the manufacturer’s instructions. For migration and invasion assays, 2 × 10^4^ cells were cultured in the upper wells of Transwell or Matrigel chambers (BD Biosciences), respectively. Cells were allowed to migrate toward 10% FBS in the bottom wells for 8–10 hours, and migrated and invaded cells were fixed in 90% ethanol and stained with 0.1% crystal violet, photographed, and counted.

### Luciferase reporter assays.

For 3′-UTR reporter assays, cells were plated in 48-well plates (5 × 10^4^ cells per well) and cotransfected with 3′-UTR luciferase reporters (10 ng), pGL3-Basic (250 ng), and miRNA mimics (50 nM) or control mimics. After 24 hours, firefly and *Renilla* luciferase activities were determined with the Dual-Luciferase Reporter Assay System (Promega).

### MS2-based RNA IP assay.

H1299 cells were cotransfected with pMS2-GFP vectors and pcDNA3.1-ACBD3 3′-UTR-WT-MS2 or pcDNA3.1-ACBD3 3′-UTR-MT-MS2 or empty vector (pcDNA3.1-MS2). After 48 hours, cells were lysed in RIPA buffer and cell lysates were subjected to RNA immunoprecipitation (RIP) experiments with an anti-GFP antibody or a control anti-IgG antibody using the Magna RIP RNA-Binding Protein Immunoprecipitation Kit (MilliporeSigma, 17-700) according to the manufacturer’s instructions. For protein detection, immunprecipitates were boiled in 1× sodium dodecyl sulfate loading buffer at 98°C for 10 minutes. The resulting samples were subjected to WB analysis. For miRNA detection, RNA in the immunoprecipitates was purified using the RNAeasy Plus kit (QIAGEN) and reverse transcribed using specific primers. The cDNA samples were subjected to quantitative reverse transcription PCR (qPCR) analysis.

### Biotin-labeled protein pulldown assay.

Recombinant SPP1 protein was biotin labeled using the DSB-X Biotin Protein Labeling Kit (Thermo Fisher Scientific) and added to the culture medium (100 ng/mL). After 4 hours, cells were harvested and lysed in RIPA buffer. The biotin-labeled SPP1 protein complex was purified using streptavidin agarose beads (Thermo Fisher Scientific), and the resulting samples were denatured at 98°C for 10 minutes and subjected to WB analysis.

### WB analysis and IP assays.

WB analysis was performed as previously described (3). For IP assays, H1299 cells were transfected with the indicated expression vectors, lysed after 48 hours in 1× RIPA buffer (Cell Signaling Technology), and incubated with antibodies at 4°C overnight. The immune complex was captured with Protein G Agarose Beads (Cell Signaling Technology), washed with 1× RIPA buffer once and 1× PBS thrice, and boiled in 1× sodium dodecyl sulfate loading buffer at 98°C for 10 minutes. The resulting samples were subjected to WB analysis.

### qPCR assays.

Total RNA was isolated using the RNeasy Mini kit (QIAGEN, 74106) and subjected to reverse transcription using qScript cDNA SuperMix (Quanta Biosciences). mRNA and miR levels were determined using SYBR Green Real-Time PCR Master Mixes (Bimake, 21203) and normalized to ribosomal protein L32 mRNA (RL32, for mRNAs) or RNU6-1 (U6, for miRs). The PCR primers used are listed in Supplemental Table 2.

### ChIP.

As previously described (21), lysates from siRNA-transfected H1299 cells were subjected to cross-linking followed by sonication and IP with anti–RNA Pol II or anti–rabbit IgG (Santa Cruz Biotechnology). Genomic DNA was eluted and purified using the MinElute Reaction Cleanup kit (QIAGEN) and subjected to qPCR. The PCR primers used are listed in Supplemental Table 2.

### BRET assay.

As previously described (32), 5,000 H1299 cells seeded in white 96-well plates were transfected with 100 ng BRET sensor using Polyplus transfection reagent according to the manufacturer’s protocol. After 24 hours, cells were washed with PBS and incubated with 50 μL modified Krebs-Ringer buffer (120 mM NaCl, 4.7 mM KCl, 0.7 mM MgSO_4_, 10 mM HEPES, pH 7.3, 10 mM D^+^ glucose, 2 mM CaCl_2_; final pH 7.4) at room temperature for 30 minutes. Forty microliters of coelenterazine h (final concentration 5 μM) in modified Krebs-Ringer buffer was added to the cells, and both Venus fluorescence and Sluc luminescence were monitored with a Synergy H1 Hybrid Multi-Mode Reader (BioTek, Agilent) customized with emission filters (540/40 nm and 475/20 nm). The plates were removed for the addition of A1 in 10 μL volume, and Venus fluorescence and Sluc luminescence were measured every 10 minutes.

### HUVEC tube formation assay.

HUVECs (1 × 10^5^ cells/well) were seeded in Matrigel-coated 12-well plates and treated with CM samples. After an 8-hour incubation, HUVECs were photographed under a phase-contrast microscope (10×), and tube-like structures were quantified from 10 randomly chosen fields.

### Flow cytometric analysis of tumor tissues.

Five weeks after subcutaneous injection of 1 × 10^6^ cancer cells into the flanks of nude mice, subcutaneous tumors were removed at necropsy and digested using collagenase I (3 mg/mL) and dispase II (4mg/mL). After single-cell suspensions were obtained, RBCs were lysed using 1× RBC lysis buffer (BioLegend) following the manufacturer instructions. The single-cell suspension was stained according to standard protocols with anti–cleaved caspase 3 (Cell Signaling Technology) and anti-CD31 (R&D Systems) antibodies. Data were acquired on a Fortessa X20 analyzer (BD Biosciences) and analyzed using FlowJo software (version 7.6, Tree Star).

### VSV-G assay.

Cells were transiently transfected with a EGFP–VSV-G (ts045) plasmid. After 24 hours, cells were transferred to a restrictive temperature of 40°C for 18 hours followed by transfer to 32°C in the presence of 100 mg/mL cycloheximide. Cells were fixed after 30 minutes or 60 minutes, and exofacial VSV-G was detected in nonpermeabilized cells by staining with IE9F9 (I14) anti–VSV-G monoclonal antibody. VSV-G trafficking to the plasma membrane was measured by the ratio of exofacial (surface) VSV-G fluorescence signal to the EGFP (total VSV-G) signal intensity (*n* = 15–20 cells per group).

### Tfn uptake and recycling assay.

Tfn endocytosis assays were performed as previously described (35). In brief, 2 × 10^4^ cells were seeded in each well on gelatin-coated 96-well plates and incubated for 24 hours. Cells were then treated with 5 μg/mL biotinylated-Tfn in assay buffer (PBS4+: PBS supplemented with 1 mM MgCl_2_, 1 mM CaCl_2_, 5 mM glucose, and 0.2% BSA) at 4°C for 10 minutes followed by transfer to 37°C for 5, 10, and 15 minutes. Cells were transferred onto ice to stop endocytosis, and unbound Tfn was removed by 3 washes with stripping buffer (0.2 M acetic acid, 0.2 M NaCl, pH 2.5). For Tfn recycling assay, cells in 96-well plates were incubated with 10 μg/mL biotinylated Tfn in PBS4+ buffer for a 30-minute pulse at 37°C and incubated in the assay buffer containing 10 mM Tris-(2-carboxyethyl)phosphine hydrochloride (TCEP) for 30 minutes at 4°C before recycling assays were performed. Cells were washed with cold PBS4+ buffer and then incubated in PBS4+ containing 2 mg/mL holo-Tfn (no. T0665, MilliporeSigma) at 37°C for the indicated durations. Cells were then immediately transferred onto ice to stop internalization. The unbound biotinylated Tfn was removed by washing with stripping buffer. Cells were then washed with cold PBS and fixed in 4% paraformaldehyde (Electron Microscopy Sciences) in PBS for 30 minutes and further permeabilized with 0.1% Triton X-100/PBS for 10 minutes. Intracellular biotinylated Tfn was assessed by streptavidin-POD (no. 11089153001, MilliporeSigma) in Q-PBS (0.2% BSA, 0.001% saponin, and 0.01% glycine). The reaction was further developed with OPD (MilliporeSigma) and then stopped by addition of 50 μL H_2_SO_4_ (5 M). The absorbance was read at 490 nm (Synergy H1 Hybrid Reader, BioTek, Agilent).

### CM transfer assays.

As previously described (3), 4 × 10^6^ cells were serum starved for 16 hours, and the supernatants (CM) were collected, filtered through a 0.45 μm filter, mixed with complete growth medium (1:1), and applied to cells. CM samples were replaced every 2 days in colony formation assays.

### Liquid chromatography–mass spectrometry analysis.

To identify PI4K2A-dependent secreted proteins, we collected and concentrated CM samples using Amicon Ultra Centrifugal Filters (MilliporeSigma) and subjected them to liquid chromatography–mass spectrometry (LC-MS) as previously described (3). To identify PI4K2A-interacting proteins by the IP/MS method, the HA-PI4K2A expression vector was stably expressed in PI4K2A-deficient H1299 cells, and cell lysates were immunoprecipitated by HA-tag antibody and isolated on Protein G beads. Proteins in the immune complex were identified by LC-MS as previously described (5). To identify PI4K2A-interacting proteins by the TurboID method, the TurboID-PI4K2A construct was transfected into H1299 cells and incubated cells in culture medium with or without 100 μM biotin. After 1 hour, cell lysates were collected, and biotinylated proteins were purified using Pierce Streptavidin Agarose Beads (Thermo Fisher Scientific). Proteins on beads were identified by LC-MS as previously described (5).

### Isolation of Golgi- and vesicle-enriched subcellular fractions.

As previously described (65), the Minute Golgi Apparatus Enrichment Kit (GO-037, Invent Biotechnologies) was used to enrich cell lysates in Golgi and vesicle fractions according to the manufacturer’s instructions.

### Cell imaging.

Cells were washed with 1× PBS 3 times and fixed using 4% paraformaldehyde for 8 minutes, permeabilized using 0.1% Triton X-100 for 8 minutes, and blocked with 3% BSA for 20 minutes. Primary antibody incubation was performed in 3% BSA for 1 hour at ambient temperature or overnight at 4°C, followed by Alexa Fluor–conjugated secondary antibodies (1:500) in 3% BSA for 1 hour at ambient temperature. Nuclei were detected by DAPI staining of cells on coverslips mounted in ProLong Gold Antifade mounting medium (Thermo Fisher Scientific). Confocal imaging was performed on an A1+ platform (Nikon Instruments) equipped with 63×/1.4 NA Oil, 100×/1.45 NA Oil, and 20×/0.75 NA Air objectives, 405/488/561 nm laser lines, GaAsP detectors, and an Okolab Top Stage Incubator. Images were processed using ImageJ (NIH) (66). A colocalization analysis (Huygens Professional) was plotted using Manders’ coefficients expressed as the percentage of red pixels that overlapped with green pixels.

### Statistics.

Unless stated otherwise, the results shown are representative of replicated experiments, and data represent the mean ± SD from triplicate samples or randomly chosen cells within a field. When performing the correlation analysis and comparing the mRNA levels of PI4Ks with EMT scores in human lung cancers, the EMT score was calculated as previously described (67). Kaplan-Meier survival data were generated using KMPlot (68). To analyze the prognostic value of a given gene signature, the average of the *z*-normalized values for the genes was evaluated across the lung compendium cohort. We used RNA-Seq data from TCGA, which includes breast-invasive carcinoma (BRCA) (*n* = 1,095); esophageal carcinoma (ESCA) (*n* = 184); kidney renal clear cell carcinoma (KIRC) (*n* = 533); liver hepatocellular carcinoma (LIHC) (*n* = 371); LUAD (*n* = 515); LUSC (*n* = 501); and ovarian serous cystadenocarcinoma (OV) (*n* = 262). Statistical evaluations were performed with GraphPad Prism 6 (GraphPad Software). An unpaired, 2-tailed Student’s *t* test was used to compare the mean values of 2 groups. ANOVA with Dunnett’s test was used to compare multiple treatments with a control. *P* values of less than 0.05 were considered statistically significant.

### Study approval.

All mouse studies were approved by the IACUC of The University of Texas–MD Anderson Cancer Center. Mice underwent standard care and were euthanized at predetermined time points or at the first signs of morbidity according to the standards set forth by the IACUC.

## Author contributions

XT conceived, designed, executed, and interpreted the molecular biology, cell biology, and in vivo experiments. GYX conceived, designed, executed and interpreted the RAB6A vesicle fission and VSV-G trafficking experiments. SW assisted XT with colony formation assays and IP experiments. LS assisted XT with the IP and WB experiments. YZ assisted XT with the luciferase reporter assay to determine the interaction between miR-34a and ACBD3 3′-UTR. XL assisted XT with the in vivo experiments. JY bred the mice for the in vivo studies. WKR directed and interpreted the mass spectrometry experiments. CJC directed and interpreted the bioinformatics analysis. JMK conceived and supervised the project and contributed to the design and interpretation of all experiments.

## Supplementary Material

Supplemental data

Supplemental table 1

Supplemental table 2

## Version 1 02/09/2023

In-Press Preview

## Version 2 04/03/2023

Electronic publication
