# Supplementary material for: EMT-activated secretory and endocytic vesicular trafficking programs underlie a vulnerability to PI4K2A antagonism in lung cancer
Source: J Clin Invest. 2023 Apr 3;133(7):e165863. doi: 10.1172/JCI165863 (PMC10065074; doi:10.1172/JCI165863)
Supplement: Supplemental data [file jci-133-165863-s177.pdf]

Supplemental figures

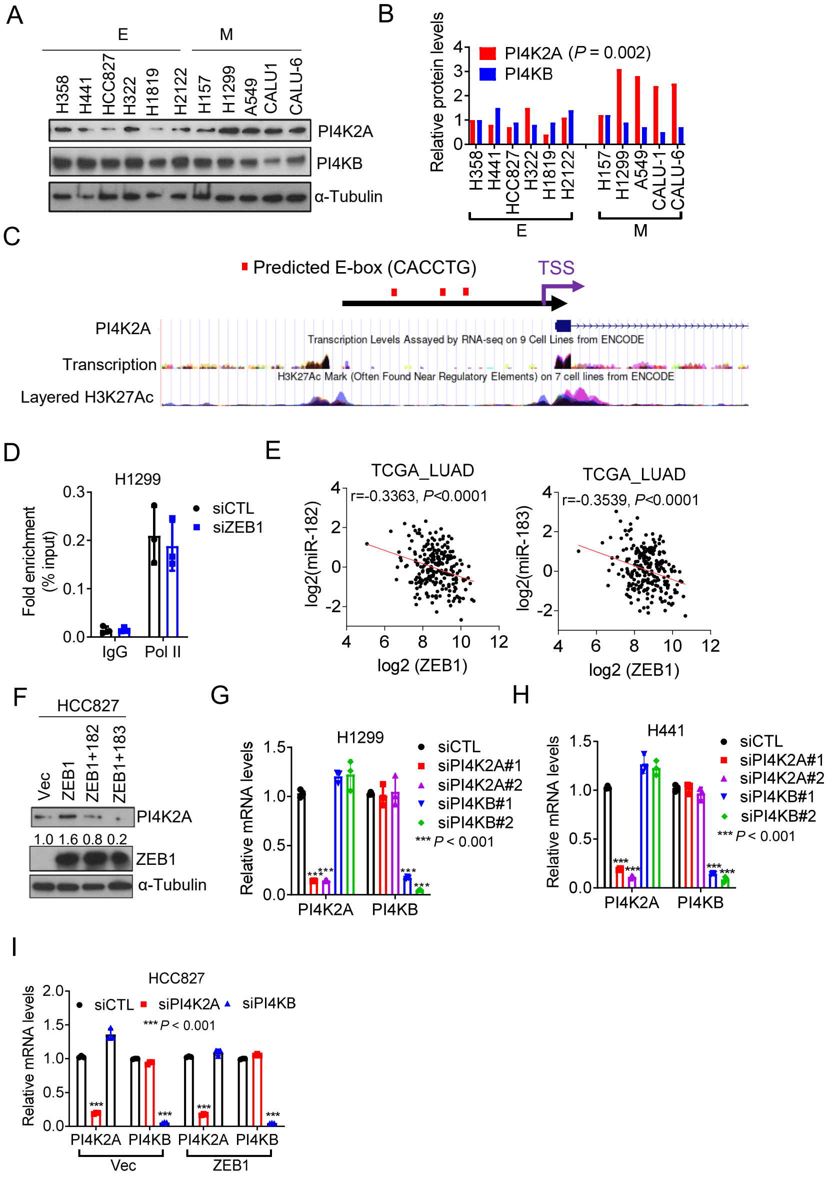

Figure S1. ZEB1 executes a PI4P-generating enzymatic switch. (A) WB analysis of PI4K2A and PI4KB in human lung cancer cell lines classified as epithelial ('E') or mesenchymal ('M'). (B) Densitometric quantification of results in (A). (C) *PI4K2A* gene locus. Locations of E-boxes in promoter region and transcription start site (TSS) (https://genome.ucsc.edu/) are indicated. (D) RNA Pol II ChIP assay on PI4K2A gene promoter. Values expressed as percentages of input (total chromatin). (E) Correlations between ZEB1 mRNA levels and miR-182 (left plot) or miR-183 (right plot) levels in tumors (dots) from TCGA LUAD cohort. (F) WB analysis of PI4K2A levels in HCC827 cells that stably express ectopic ZEB1 (ZEB1) or empty vector (Vec) and were transfected with miR mimics. Relative densitometric values under gels. (G-I) qPCR analysis of PI4K2A and PI4KB mRNA levels in H1299 (E), H441 (F), and HCC827 (G) transfectants.

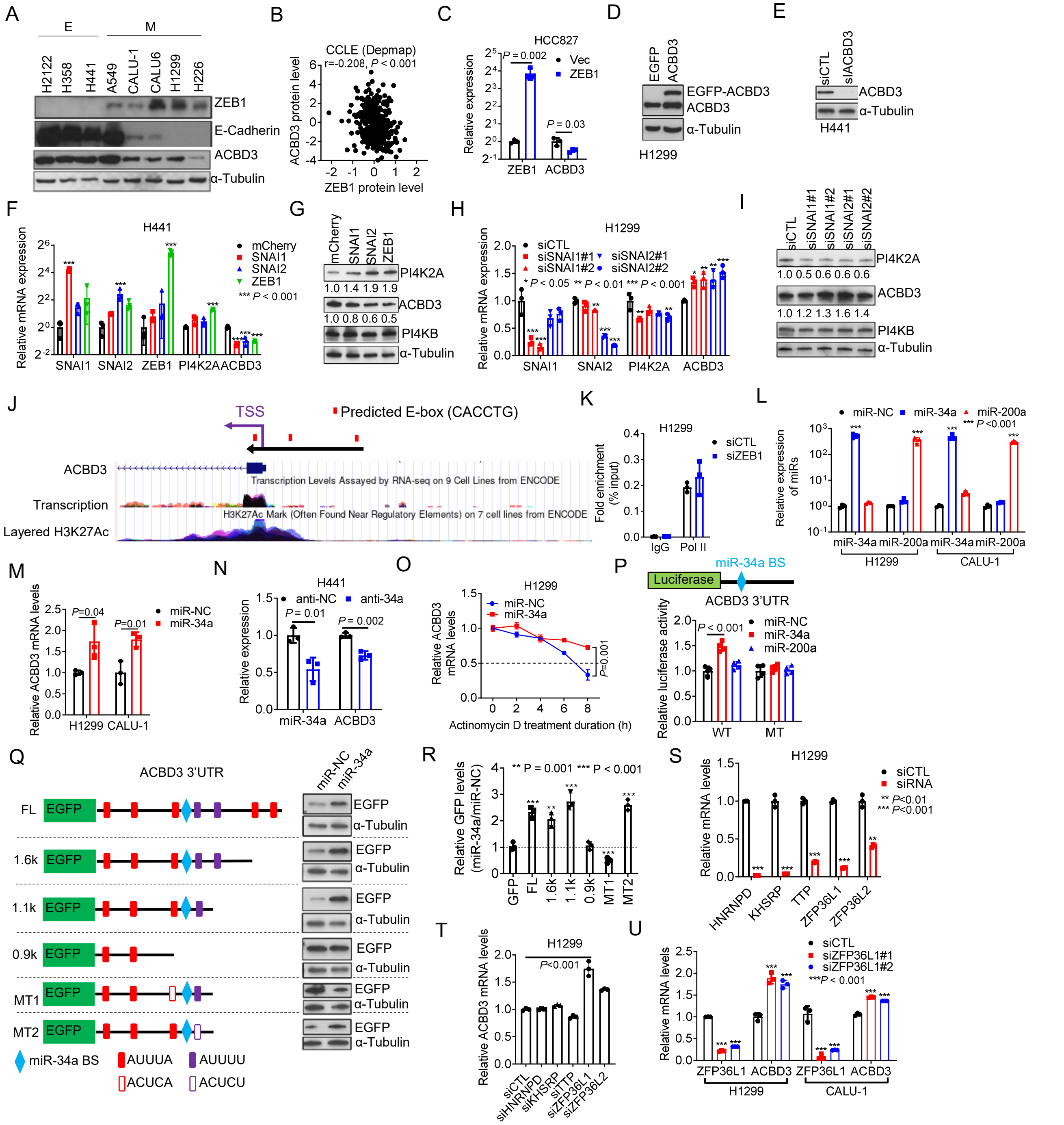

Figure S2. PI4K2A and ACBD3 are regulated by EMT-TFs. (A) WB analysis of ACBD3 levels in lung cancer cell line panel. E-cadherin included as an epithelial cell marker. (B) Correlation between ACBD3 and ZEB1 protein levels in lung cancer cell lines (dots) in CCLE (www.broadinstitute.org/ccle). (C) qPCR analysis of ZEB1 and ACBD3 mRNA levels in HCC827_ZEB1 cells (ZEB1) and HCC827_vector cells (Vec). (D and E) WB analysis of ACBD3 protein levels in H1299 (D) and H441 (E) transfectants. (F-I) qPCR analysis (F, H) and WB analysis (G, I) of PI4K2A, PI4KB, and PI4KB in siRNA-transfected H441 cells. Relative densitometric values under gels. (J) *ACBD3* gene locus. Locations of predicted E-boxes in promoter region and transcription start site (TSS) (https://genome.ucsc.edu/) are indicated. (K) RNA Pol II ChIP assays on *ACBD3* gene promoter. Values expressed as percentages of input (total chromatin). Data are the mean ± SD from a single experiment incorporating biological replicate samples (n = 3, unless otherwise indicated) and are representative of at least 2 independent experiments. Two-tailed Student's t test for 2-group comparisons; 1-way ANOVA test for multiple comparisons. (L) qPCR analysis to confirm ectopic expression of miR mimics. Negative control mimic (miR-NC). (M) qPCR analysis of ACBD3 mRNA levels in miR-34a

mimic-transfected cells. (N) qPCR analysis of miR34a and ACBD3 mRNA levels in H441 cells transfected with antagomiR-34a (anti-34a) or negative control (anti-NC). (O) qPCR analysis of ACBD3 mRNA levels in miR mimic-transfected cells treated with 5 μg/ml actinomycin D. (P) ACBD3 3'-UTR reporter assays. H1299 cells co-transfected with miR mimics and wild-type (WT) or mutant (MT) ACBD3 3′-UTR reporters lacking the miR-34a binding site (n = 4 replicates per condition). (Q) Schematic illustration of full-length (FL) and truncated constructs shows locations of wild-type (solid icons) and mutant (empty icons) binding sites for RNA-binding proteins (AUUUA, AUUUU). WB analysis of ACBD3 3'-UTR-driven EGFP reporters in miR mimic-transfected H1299 cells. (R) Densitometric analysis of (Q). (S) qPCR analysis of target genes in siRNA-transfected H1299 cells. (T, V) qPCR analysis of ACBD3 mRNA levels in siRNA-transfected cells. Data are the mean ± SD from a single experiment incorporating biological replicate samples (n = 3, unless otherwise indicated) and are representative of at least 2 independent experiments. Two-tailed Student's t test for 2-group comparisons; 1-way ANOVA test for multiple comparisons.

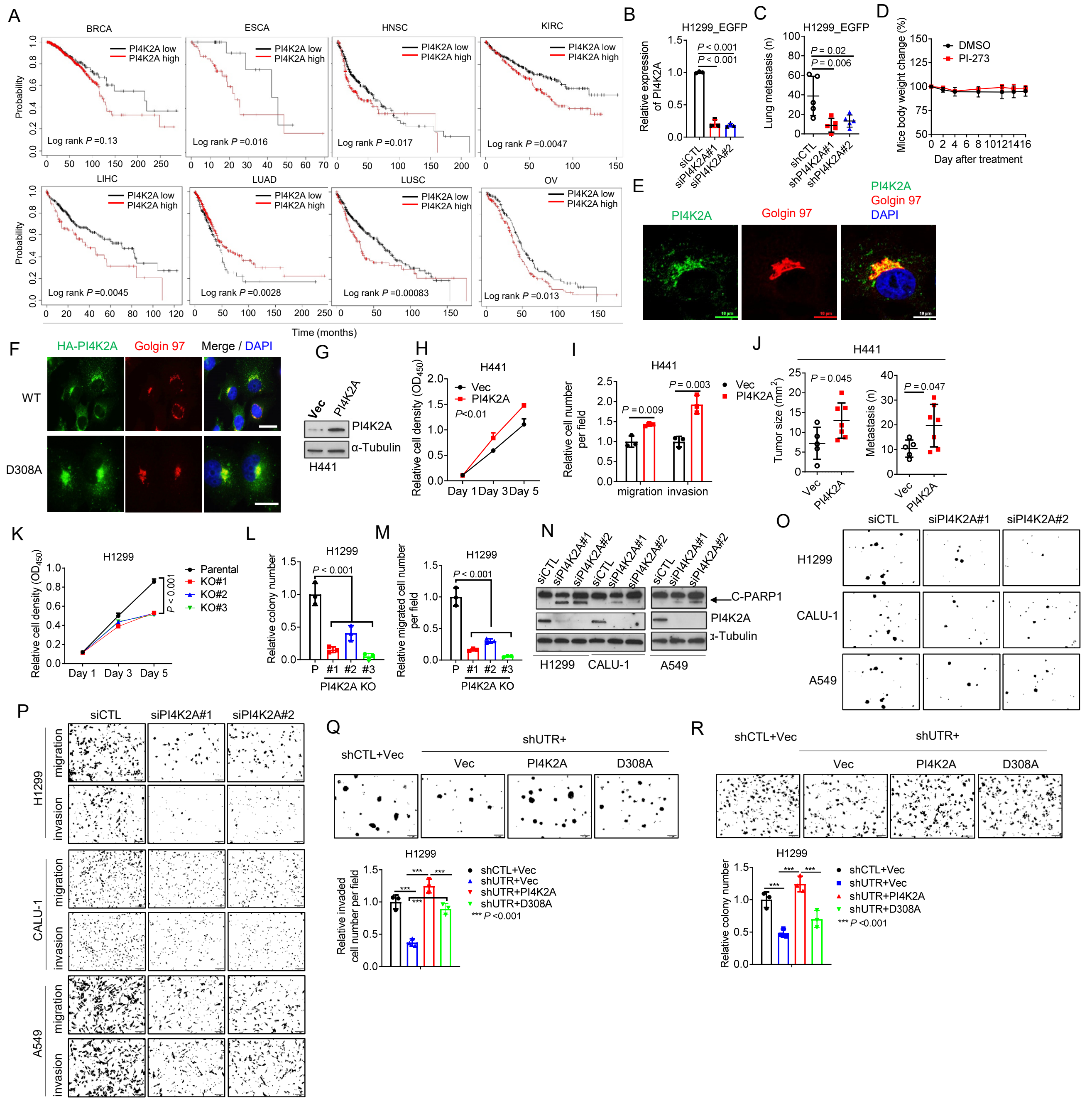

Figure S3. PI4K2A drives lung cancer progression. (A) Kaplan-Meier survival analysis of TCGA cohorts based on PI4K2A mRNA levels above (high) or below (low) the median value in each cohort. See methods for tumor type abbreviations and sample sizes in each cohort. (B) qPCR analysis to confirm target gene depletion in PI4K2A shRNA-transfected H1299 cells. (C) Lung metastasis numbers per mouse (dots) injected by tail vein with green fluorescent protein (GFP)–tagged H1299 cells generated in (B). (D) Mouse body weights during PI-273 or DMSO treatment normalized to day 0. (E) Single channel and merged confocal micrographs of H1299 cells demonstrate localization of endogenous PI4K2A in the Golgi. Cells co-stained with α-PI4K2A and α-Golgin-97 antibodies. (F) Single channel and merged confocal micrographs demonstrate localization of ectopic PI4K2A in the Golgi. H1299 cells transfected with HA-tagged wild type or mutant (D308A) PI4K2A and co-stained with α-HA and α-Golgin-97 antibodies. (G) WB analysis of PI4K2A levels in H441 cells stably transfected with PI4K2A or empty vector (Vec). (H) Relative densities of cells described in (G) in monolayer culture. (I) Boyden chamber migration and invasion assays on cells described in (G). (J) Orthotopic lung tumor sizes (left plot) and mediastinal and contralateral lung metastasis numbers (right plot) per mouse (dots). Tumors generated by intra-thoracic injection of cells described in (G). (K) Relative densities of parental and PI4K2A knockout (KO) H1299 cells in monolayer culture. KO clones (#1-3). (L) Colony formation in soft agar. Values for PI4K2A KO clones expressed relative to parental cells. (M) Boyden chamber migration assays. Values for PI4K2A KO clones expressed relative to parental cells. (N) WB analysis of cleaved PARP1 (C-PARP1) to detect apoptosis in siRNA-transfected cells. (O) Soft agar colonies generated by siRNA-transfected cells. (P) Migrated and invaded siRNA-transfected cells in Boyden chambers. (Q, R) Soft agar colony assays (Q) and Boyden chamber migration assays (R) on H1299 cells subjected to shRNA-mediated PI4K2A depletion (shUTR) and reconstituted with wild-type (PI4K2A) or enzyme-dead mutant (D308A) PI4K2A. Control shRNA (shCTL). Empty vector (Vec). Data are the mean ± SD from a single experiment incorporating biological replicate samples ($n = 3$, unless otherwise indicated) and are representative of at least 2 independent experiments. Two-tailed Student's t test for 2-group comparisons; 1-way ANOVA test for multiple comparisons.

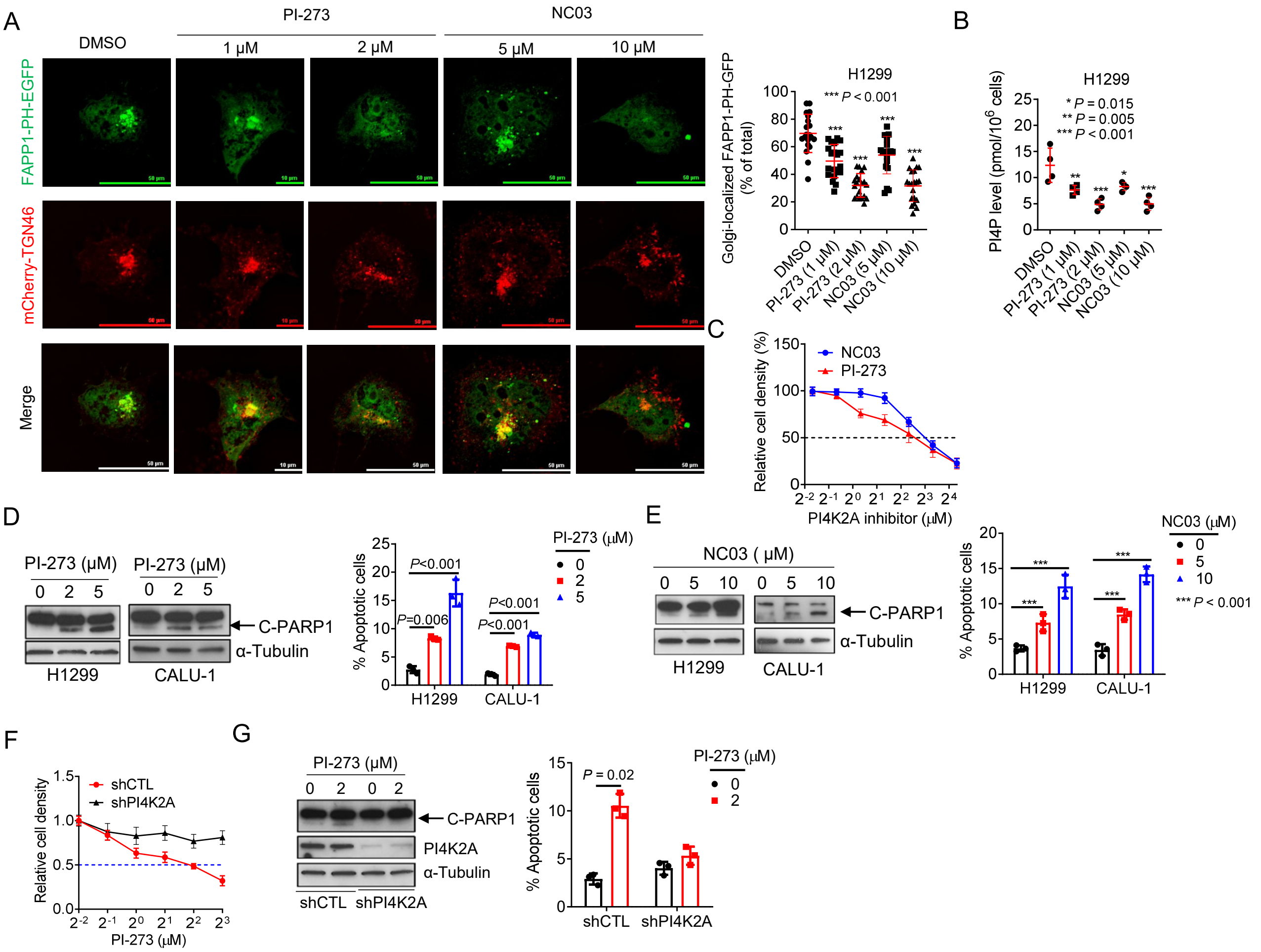

Figure S4. PI4K2A inhibitors induce apoptosis in mesenchymal lung cancer cells (A) Single channel and merged confocal micrographs of H1299 cells co-transfected with a FAPP1-PH-EGFP reporter and a Golgi marker expression vector (mCherry-TGN48) and treated with PI4K2A inhibitor (PI-273 or NC03) at different doses. The percentages of total FAPP1-PH-GFP signal that localized in the Golgi per cell was quantified (dot plot). PI4K2A inhibitors decreased PI4K2A activity as demonstrated by loss of Golgi-localized FAPP1 reporter. (B) PI4P ELISA on H1299 cells treated with indicated doses of PI-273 or NC03 (n=4 replicates [dots] per condition). (C) Relative densities of H1299 cells treated with different doses of PI-273 or NC03. (D, E) Apoptosis quantified by WB analysis of cleaved PARP1 (C-PARP1) (gels) and flow cytometric analysis of Annexin V/PI-stained cells (graphs) following treatment with different doses of PI-273 (D) or NC03 (E). (F, G) Relative cell density assays (F) and apoptosis assays (G) on shRNA-transfected H1299 cells treated with PI-273 show that PI4K2A depletion mitigated the effects of PI-273. Data are the mean ± SD from a single experiment incorporating biological replicate samples (n = 3, unless otherwise indicated) and are representative of at least 2 independent experiments. Two-tailed Student’s t test for 2-group comparisons; 1-way ANOVA test for multiple comparisons.

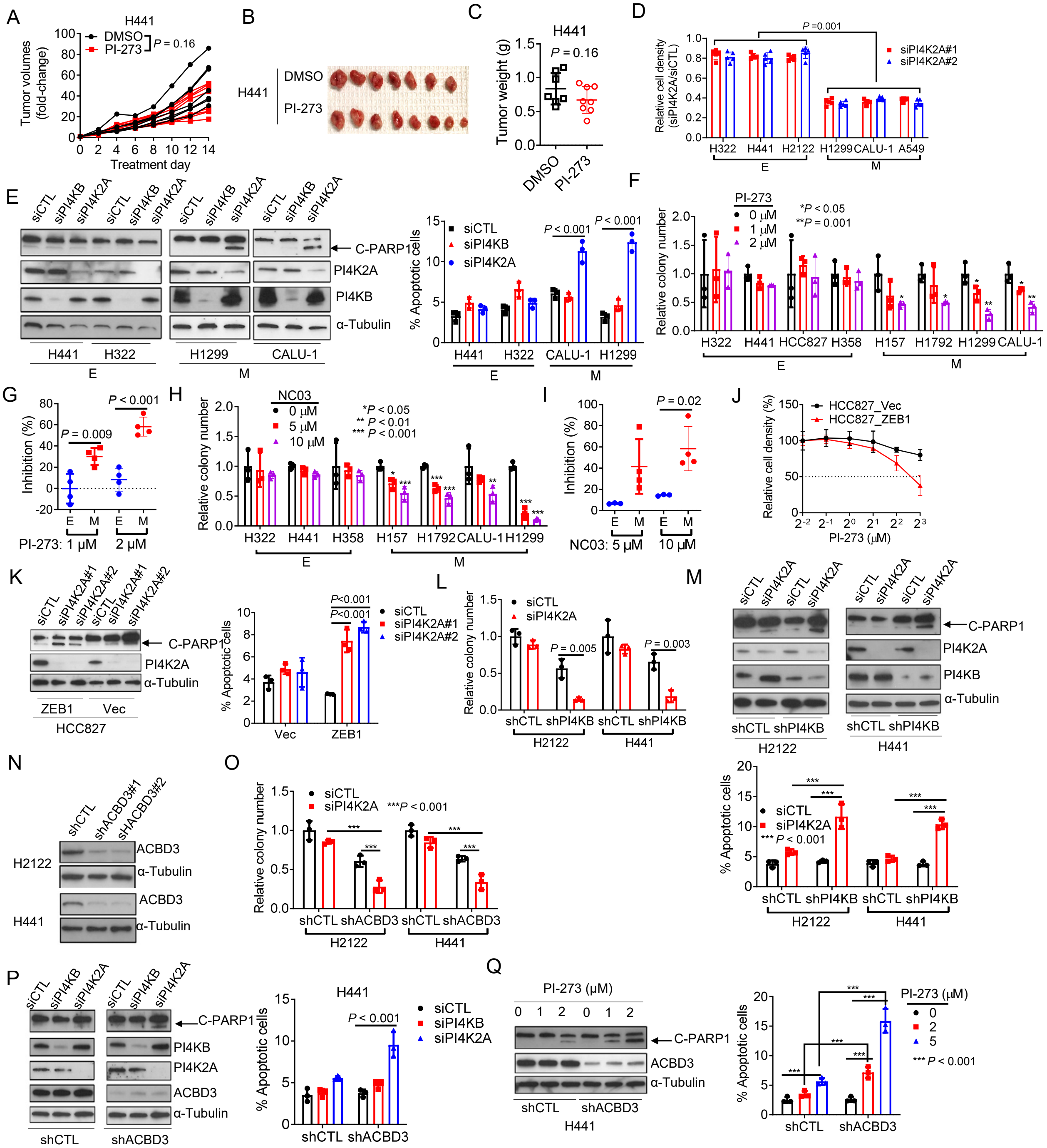

Figure S5. Sensitivity to PI4K2A inhibition is higher in mesenchymal ('M') than epithelial ('E') cells. (A) Daily subcutaneous tumor volume measurements (dots) in nude mice treated with PI-273 or vehicle. (B, C) Tumor tissues removed at necropsy in (A) were imaged (B) and weighed (C). (D) Relative densities of siRNA-transfected 'E' and 'M' cells in monolayer culture. Results normalized to siCTL. (E) Apoptosis in siRNA-transfected 'E' and 'M' cells detected by WB analysis C-PARP1 (gels) and flow cytometric analysis of Annexin V/PI staining (graph). (F) Relative colony numbers generated by 'E' and 'M' cells treated with PI273 or vehicle (0 μM). (G) Results from (F) expressed as the percentage inhibitions of colony formation induced by drug relative to vehicle. (H) Relative colony numbers generated by 'E' and 'M' cells treated with NC03 or vehicle (0 μM). (I) Results from (H) expressed as the percentage reductions in colony formation induced by drug relative to vehicle. (J) Relative densities of HCC827_ZEB1 cells and HCC827_vector cells treated with PI-273 in monolayer culture demonstrate that ectopic ZEB1 expression enhances sensitivity to PI-273. (K) Apoptosis assays on HCC827_ZEB1 cells and HCC827_vector cells show that ectopic ZEB1 expression

enhances apoptosis induced by PI4K2A depletion. (L, M) Colony formation assays (L) and apoptosis assays (M) on PI4KB-deficient or -replete ‘E’ cells subjected to siRNA-mediated PI4K2A depletion show that PI4KB depletion enhances sensitivity to PI4K2A inhibition in ‘E’ cells. (N) WB analysis to confirm target gene depletion in shRNA-transfected cells. (O-Q) Relative colony numbers generated by ACBD3-deficient or -replete ‘E’ cells on plastic following siRNA-mediated PI4K2A depletion show that ACBD3 depletion enhances sensitivity to PI4K2A inhibition in ‘E’ cells. (P, Q) Apoptosis assays on ACBD3 shRNA-transfected ‘E’ cells subjected to siRNA-mediated PI4K2A depletion (P) or PI-273 treatment (Q) show that ACBD3 depletion enhances sensitivity to PI4K2A inhibition in ‘E’ cells. Data are the mean ± SD from a single experiment incorporating biological replicate samples (n = 3, unless otherwise indicated) and are representative of at least 2 independent experiments. Two-tailed Student’s t test for 2-group comparisons; 1-way ANOVA test for multiple comparisons.

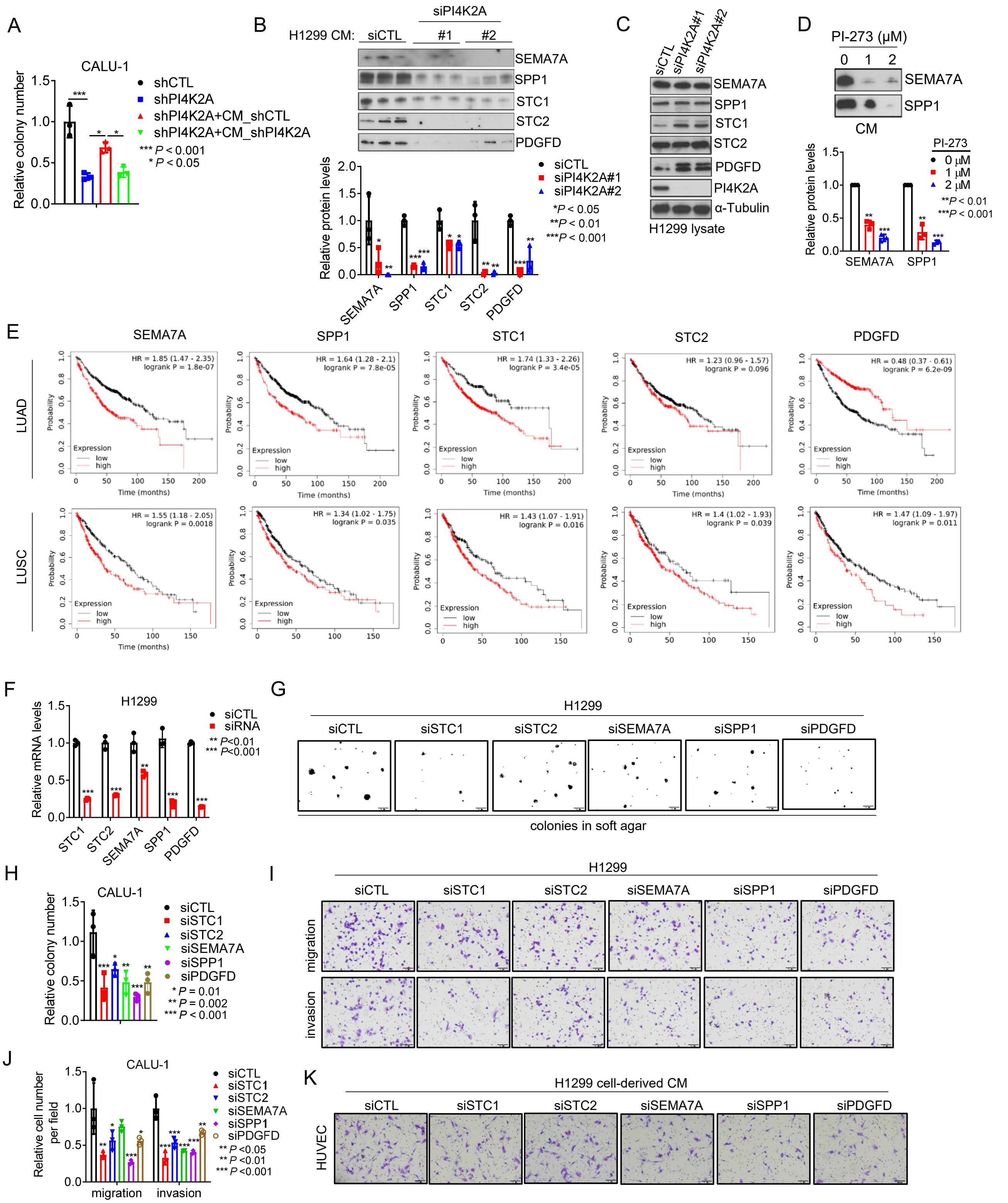

Figure S6. PI4K2A activates a pro-metastatic secretome. (A) Soft agar colony formation following treatment of shRNA-transfected CALU-1 cells with conditioned medium (CM) samples from shRNA-transfected CALU-1 cells (+CM-shCTL or -shPI4K2A). Values expressed relative to shCTL. (B) WB analysis of CM samples from siRNA-transfected H1299 cells. Results validate that proteins identified by LC-MS analysis of CM samples are secreted in a PI4K2A-dependent manner. Control siRNA (siCTL). Densitometric quantification normalized to siCTL (bar graph). (C) WB analysis of lysates from siRNA-transfected cells. Results demonstrate no effect of siRNAs on the intracellular levels of secreted proteins. (D) WB analysis of CM samples from PI-273-treated cells shows that proteins identified by LC-MS analysis of CM samples are

secreted in a PI4K2A-dependent manner. Densitometric quantification normalized to siCTL (bar graph). (E) Kaplan-Meier survival analysis of the TCGA LUAD and LUSC cohorts based on mRNA levels above (high) or below (low) the median values in those cohorts. (F) qPCR analysis of target gene mRNA levels in siRNA-transfected H1299 cells. (G, H) Soft agar colonies generated by siRNA-transfected H1299 cells were images (G) and quantified (H). (I, J) Migrated and invaded siRNA-transfected H1299 cells in Boyden chambers were imaged (I) and quantified (J). Values normalized to siCTL. (K) Migrated HUVECs in Boyden chambers. CM samples from siRNA-transfected H1299 cells added to lower chamber. Data are the mean ± SD from a single experiment incorporating biological replicate samples (n = 3, unless otherwise indicated) and are representative of at least 2 independent experiments. Two-tailed Student's t test for 2-group comparisons; 1-way ANOVA test for multiple comparisons.

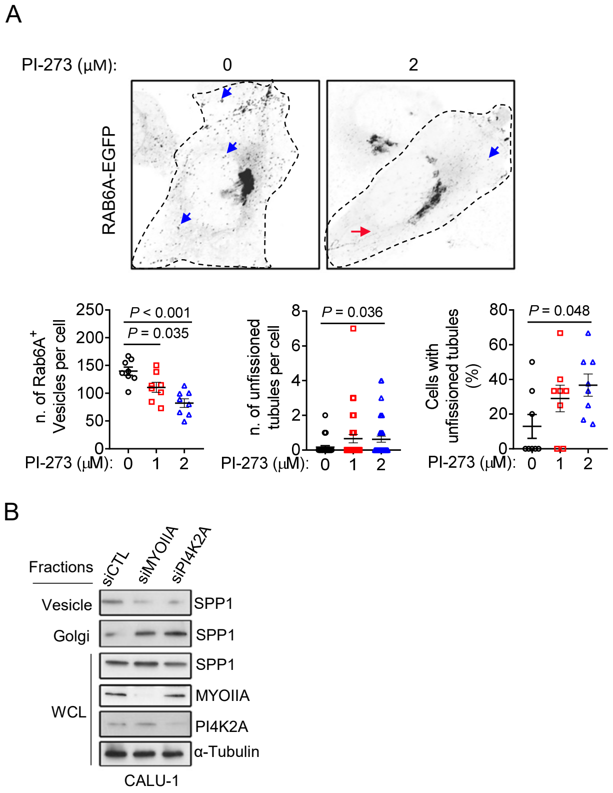

Figure S7. PI4K2A drives anterograde vesicular trafficking. (A) Contrast-adjusted confocal micrographs of Rab6A+ vesicles (blue arrows) and unfissioned Rab6A+ tubules (red arrows) emerging from the Golgi. Cell boundary (dotted lines). Results quantified per cell (dot plots). (B) WB analysis of SPP1 in whole cell lysates (WCL) or enriched subcellular fractions from siRNA-transfected CALU-1 cells. Data are the mean ± SD from a single experiment incorporating biological replicate samples (n = 3, unless otherwise indicated) and are representative of at least 2 independent experiments. 1-way ANOVA test for multiple comparisons.

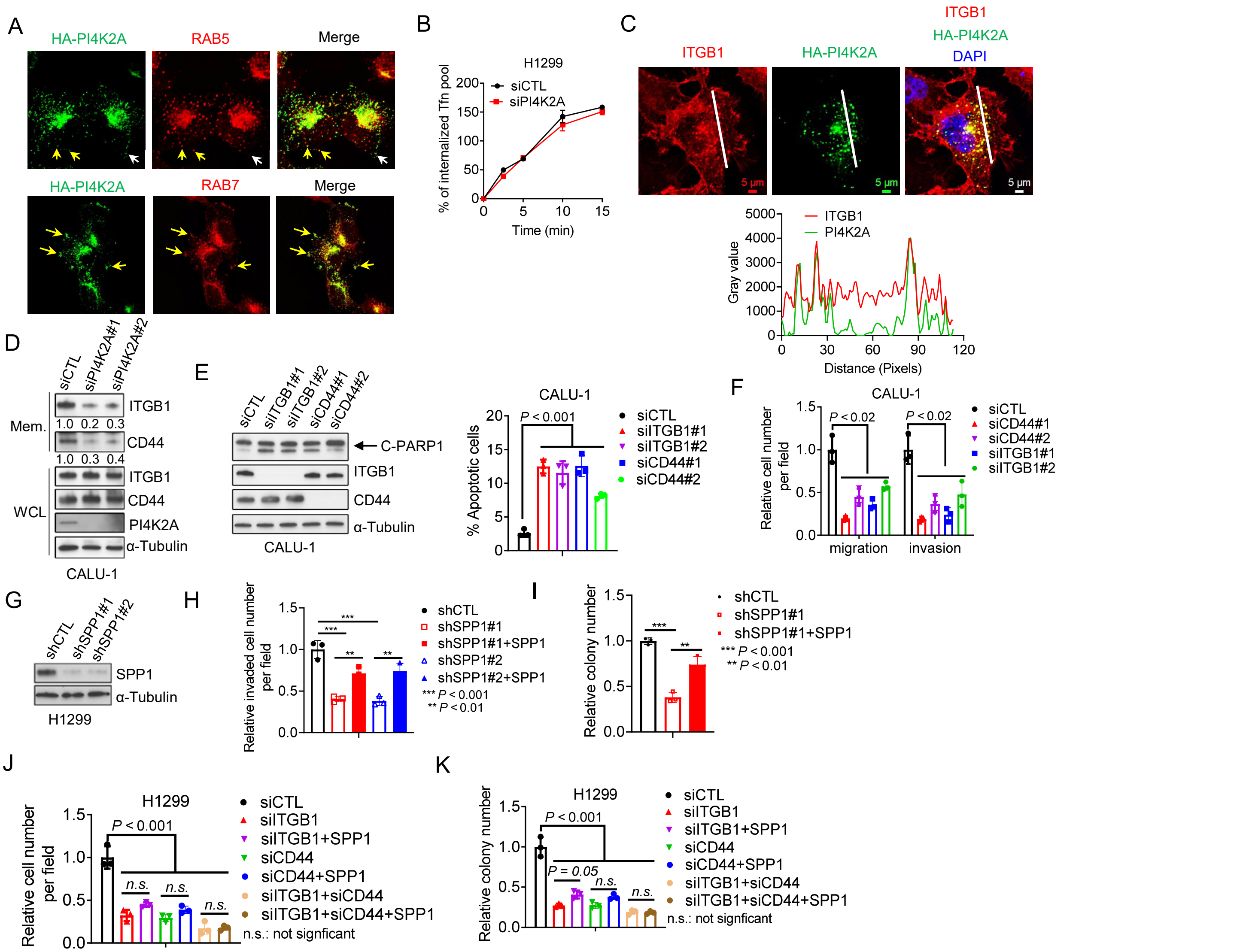

Figure S8. PI4K2A-dependent endocytic recycling activates an SPP1-dependent autocrine loop. (A) Single channel and merged confocal micrographs of H1299 cells that were transfected with HA-tagged PI4K2A and co-stained with α-HA and α-RAB5 (top panels) or α-RAB7 (lower panels) antibodies. RAB5$^+$ and RAB7$^+$ vesicles that contain PI4K2A are marked (arrows). (B) Biotinylated Tfn endocytosis rates in siRNA-transfected H1299 cells. The percentage of total biotinylated Tfn that was internalized was calculated for each sample (n = 4 samples per condition). (C) Single channel and merged confocal micrographs of H1299 cells that were transfected with HA-tagged PI4K2A and co-stained with α-HA and α-ITGβ1 antibodies. Line graph illustrates ITGβ1 and PI4K2A signal intensities (Y-axis) plotted on lines drawn from the plasma membrane inwards (X-axis) in fixed cells. (D) WB analysis of cell membrane-enriched fractions (Mem.) and whole cell lysate (WCL). Densitometric values under gels. (E) WB analysis of cleaved PARP1 (C-PARP1) (gel) and flow cytometric analysis of Annexin V/PI-stained cells (graph) to quantify apoptosis in siRNA-transfected H1299 cells. (F) Boyden chamber migration and invasion assays on siRNA-transfected cells. (G) WB analysis of target protein levels in shRNA-transfected H1299 cells. (H, I) Boyden chamber invasion assay (H) and soft agar colony formation assay (I) on H1299 cells that were transfected with SPP1 shRNAs and treated with or without recombinant SPP1. (J, K) Boyden chamber invasion assay (J) and soft agar colony formation assay (K) on H1299 cells that were transfected with ITGB1 or CD44 siRNAs and treated with or without recombinant SPP1. Data are the mean ± SD from a single experiment incorporating biological replicate samples (n = 3, unless otherwise indicated) and are representative of at least 2 independent experiments. 1-way ANOVA test for multiple comparisons.

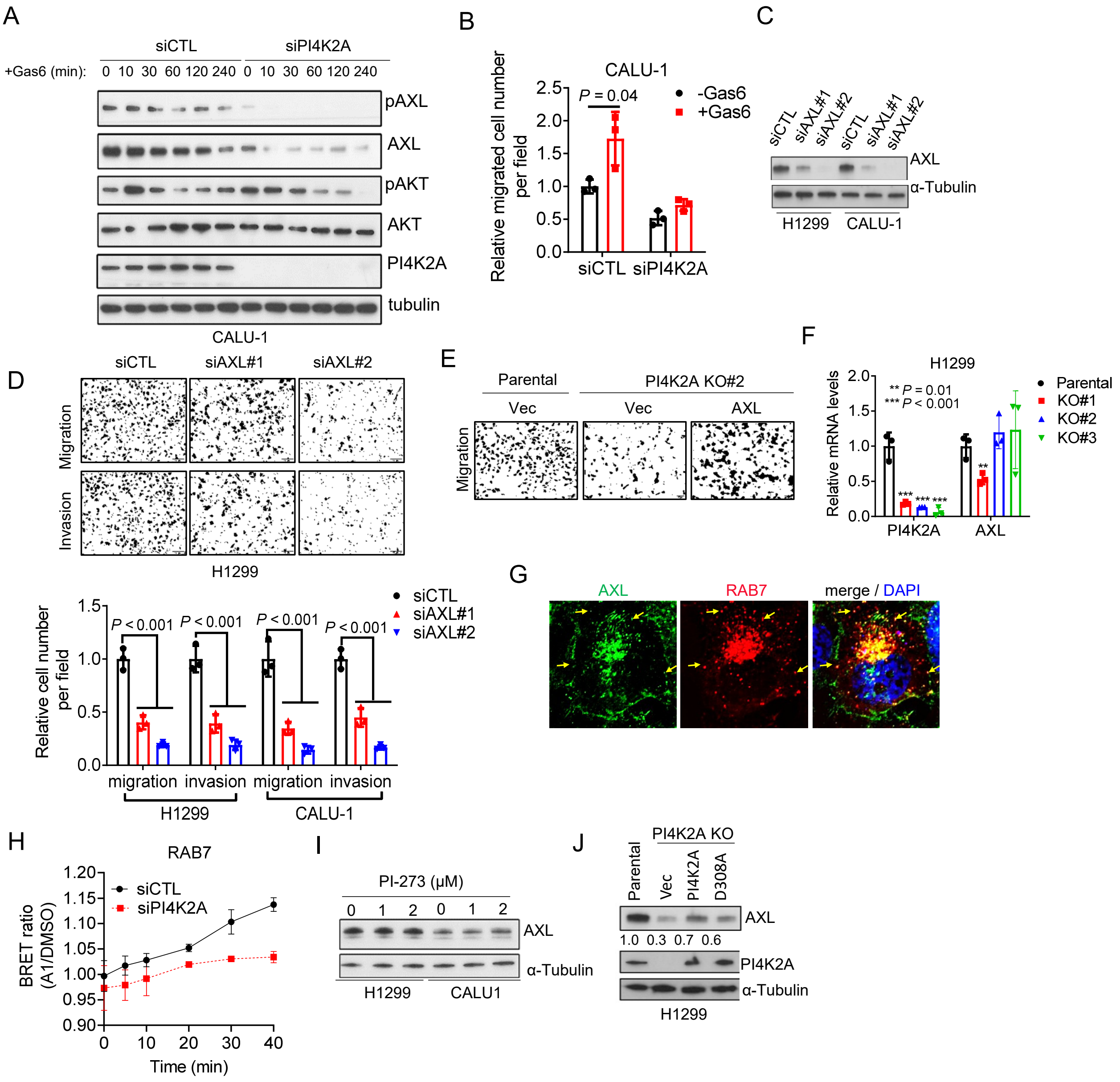

Figure S9. PI4K2A stabilizes AXL protein. (A) WB analysis of phosphorylated (p) or total protein levels in siRNA-transfected CALU-1 cells treated with or without (0) AXL ligand Gas6 for the indicated time points. (B) Boyden chamber migration assays on siRNA-transfected CALU-1 cells treated with (+) or without (-) Gas6. (C) WB analysis of target protein levels in siRNA-transfected cells. (D) Boyden chamber migration and invasion assays on siRNA-transfected cells. Results expressed relative to siCTL. (E) Boyden chamber migration assays on H1299 transfectants. (F) qPCR analysis of AXL mRNA levels in parental and PI4K2A KO clones. (G) Single channel and merged confocal micrographs of H1299 cells co-stained with α-AXL and α-RAB7 antibodies. $RAB7^+$ vesicles containing AXL are marked (arrows). (H) BRET assay to detect PI4P in $RAB7^+$ endosomes. Normalized BRET values based on ratio of values from GSKA1-/DMSO-treated cells (n=4 replicates for each condition). (I) WB analysis of AXL and PI4K2A levels in H1299 and CALU-1 cells treated with different doses of PI-273. (J) WB analysis of PI4K2A KO H1299 cells reconstituted with wild-type or enzyme-dead mutant (D308A) PI4K2A. Empty vector (Vec). Densitometric values under gels. Data are the mean ± SD from a single experiment incorporating biological replicate samples (n = 3, unless otherwise indicated) and are representative of at least 2 independent experiments. 1-way ANOVA test for multiple comparisons.
