# Supplementary material for: EMT-activated secretory and endocytic vesicular trafficking programs underlie a vulnerability to PI4K2A antagonism in lung cancer
Source: J Clin Invest. 2023 Apr 3;133(7):e165863. doi: 10.1172/JCI165863 (PMC10065074; doi:10.1172/JCI165863)
Supplement: Supplemental table 1 [file jci-133-165863-s178.pdf]

Table S1. Primer sequences.

qPCR primers		
**Gene**	**Forward (5′-3′)**	**Reverse (5′-3′)**
PI4K2A	CGAGGCAATGACAACTGGCTGA	GCCACCTTGATAACAGGCTCCT
PI4KB	TGGTCGGTGGATGACATAGGCG	CTGGTGATGCTGTCCACAGAGA
ACBD3	TCAGTGAGTCCAGCGATGACGA	GGCACAATCTCATCCAGCAAAGG
ZFP36L1	CCCTTTGAGGAAAACGGTGCCT	GCAAAAGCCGATGGTGTGGAAG
HNRNPD	GCCAAGGTTACGGTGGTTATGG	TGATGACCACCTCGCCTGGATA
KHSRP	GTGGACAAACCTCTCCGCATCA	GCCAATCCGAGATCCGTACTCA
TTP	GCTATGTCGGACCTTCTCAGAG	CCTGGAGGTAGAACTTGTGACAG
ZFP36L2	GTACGGCGAAAAGTGCCAGTTC	AGCCGATGGTATGAAAGGTGCG
STC1	GCAGGAAGAGTGCTACAGCAAG	CATTCCAGCAGGCTTCGGACAA
STC2	GCATGACTTTTCTGCACAACGCT	GGCTTATGCAGCCGAACCTGTG
SEMA7A	CTTCTTCCGAGAGGACAATCCTG	GTGTTCCACTTGGAGACTGACAG
SPP1	CGAGGTGATAGTGTGGTTTATGG	GCACCATTCAACTCCTCGCTTTC
PDGFD	GCGGCTTCACTCTCAGGAGAAT	CTTGTGTCCACACCATCGTCCT
AXL	GTTTGGAGCTGTGATGGAAGGC	CGCTTCACTCAGGAAATCCTCC
ZEB1	GGCATACACCTACTCAACTACGG	TGGGCGGTGTAGAATCAGAGTC
SNAI1	TGCCCTCAAGATGCACATCCGA	GGGACAGGAGAAGGGCTTCTC
SNAI2	ATCTGCGGCAAGGCGTTTTCCA	GAGCCCTCAGATTTGACCTGTC
ChIP-ACBD3 promoter	CAAAGTCACTGAATTTCTCTGCA	TCGACGGACACCTCGAGT
ChIP-PI4K2A promoter	TCCAGTAAGCCAATTGGAAGCT	CGCGCTCCACACCACT
**Clone primers**		
**Primers for 3’-UTR cloning (pRL-neo)**		
PI4K2A 3’-UTR	GCTCTAGACTCCAGAGGCAGGCAGAG	AAGGAAAAAAGCGGCCGCAGCTGCCCCTCCAGACA
PI4K2A 3’-UTR-MT182	CCACACCTTTCTTTTGGGTTTATCCGACGCCTCCTCCATCTCCCAT	ATGGGAGATGGAGGAGGCGTCGGATAAACCCAAAAGAAAGGTGTGG
PI4K2A 3’-UTR-MT183	GCTCTAGACTCCAGAGGCAGGCAGAG	AAGGAAAAAAGCGGCCGC AGCTGCCCCTCCAGACACAGAAGACGCTCTTTTTACTCCCTCCTGAGGC
ACBD3 3’-UTR	GCTCTAGACTAGATAAAAATGTTGTTACAAAGTCTGGA	AAGGAAAAAAGCGGCCGCTCGCACTTGTACTGTACTAATCAACAG
ACBD3 3’-UTR-MT34a	GAAGTAACATTGCACTAATATGGAAATCTCAGGCTGAGACAGTCTATTTTCTTTTAATTTGTTACTAC	GTAGTAACAAATTAAAAGAAAATAGACTGTCTCAGCCTGAGATTTCCATATTAGTGCAATGTTACTTC
**Primers for 3’-UTR cloning (pEGFP-C3)**		
ACBD3 3’-UTR-FL	CCGCTCGAGTGACTAGATAAAAATGTTGTTACAAAGTCTGGA	CGGGATCCTCGCACTTGTACTGTACTAATCAACAG
ACBD3 3’-UTR-1.6k	CCGCTCGAGTGACTAGATAAAAATGTTGTTACAAAGTCTGGA	CGGGATCCACAGTATTTTTCTCATAAAAAAAAAATCCA
ACBD3 3’-UTR-1.1k	CCGCTCGAGTGACTAGATAAAAATGTTGTTACAAAGTCTGGA	CGGGATCCTTAAGCATGGGCATATGTATTTCC
ACBD3 3’-UTR-0.9k	CCGCTCGAGTGACTAGATAAAAATGTTGTTACAAAGTCTGGA	CGGGATCCGATTTTATGTATTTTAATTCAGTAGGTGCC
ACBD3 3’-UTR-MT1	GCACCTACTGAATTAAAATACATAAAATCACTCAAATATAATTCAGCATATGGGAAGTAACA	TGTTACTTCCCATATGCTGAATTATATTTGAGTGATTTTATGTATTTTAATTCAGTAGGTGC
ACBD3 3’-UTR-MT2	GAAATCACTGCCAGAGACAGTCTACTCTCTTTTAATTTGTTACTACTTAGTCACACTTAG	CTAAGTGTGACTAAGTAGTAACAAATTAAAAGAGAGTAGACTGTCTCTGGCAGTGATTTC
**Primers for gene expression (pcDNA3.1)**		
PI4K2A (HA tag)	CCGCTCGAGATGTACCCATACGATGTTCCAGATTACGCTATGGACGAGACGAGCCC	GCTCTAGACTACCACCATGAAAAGAAGGG
D308A (HA tag)	CTTACATCATCCGCAACACTGCTCGAGGCAATGACAACTGG	CCAGTTGTCATTGCCTCGAGCAGTGTTGCGGATGATGTAAG
PI4K2A (1-479) (MYC tag)	CCGCTCGAGATGGAACAAAAACTCATCTCAGAAGAGGATCTGGACGAGACGAGCCCAC	CGGGGTACCCTACCACCATGAAAAG
PI4K2A (1-266) (MYC tag)	CCGCTCGAGATGGAACAAAAACTCATCTCAGAAGAGGATCTGGACGAGACGAGCCCAC	CGGGGTACCCTAGCCTTCAACAAAGAG
PI4K2A (79-266) (MYC tag)	CCGCTCGAGATGGAACAAAAACTCATCTCAGAAGAGGATCTGGCGCAGGCCCAGGCTC	CGGGGTACCCTAGCCTTCAACAAAGAG

PI4K2A (79-479) (MYC tag)	CCGCTCGAGATGGAACAAAAACTCATCTCAGAAGAG GATCTGGCGCAGGCCCAGGCTC	CGGGGTACCCTACCACCATGAAAAG
PI4K2A (267-479) (MYC tag)	CCGCTCGAGATGGAACAAAAACTCATCTCAGAAGAG GATCTGTACAAAGATGCAGACT	CGGGGTACCCTAGCCTTCAACAAAGAG
**Primers for gene expression (pEGFP-C3)**		
ACBD3	GGAATTCATACGTGGCTGCCGTCTG	GGGGTACCTCTAGTATAATAGACTCTGTAGTAGACTGATT TTGA
**Primers for gene expression (Sluc-P4M2X-T2A-Venus)**		
RAB6A	CCCAAGCTTATGTCCACGGGCGGAG	GCTCTAGATTAGCAGGAACAGCCTCCTTC
